# Supplementary figures and images for: SARS-CoV-2 RNA detection on environmental surfaces in COVID-19 wards
Source: PLoS One. 2023 May 25;18(5):e0286121. doi: 10.1371/journal.pone.0286121 (PMC10212077; doi:10.1371/journal.pone.0286121)

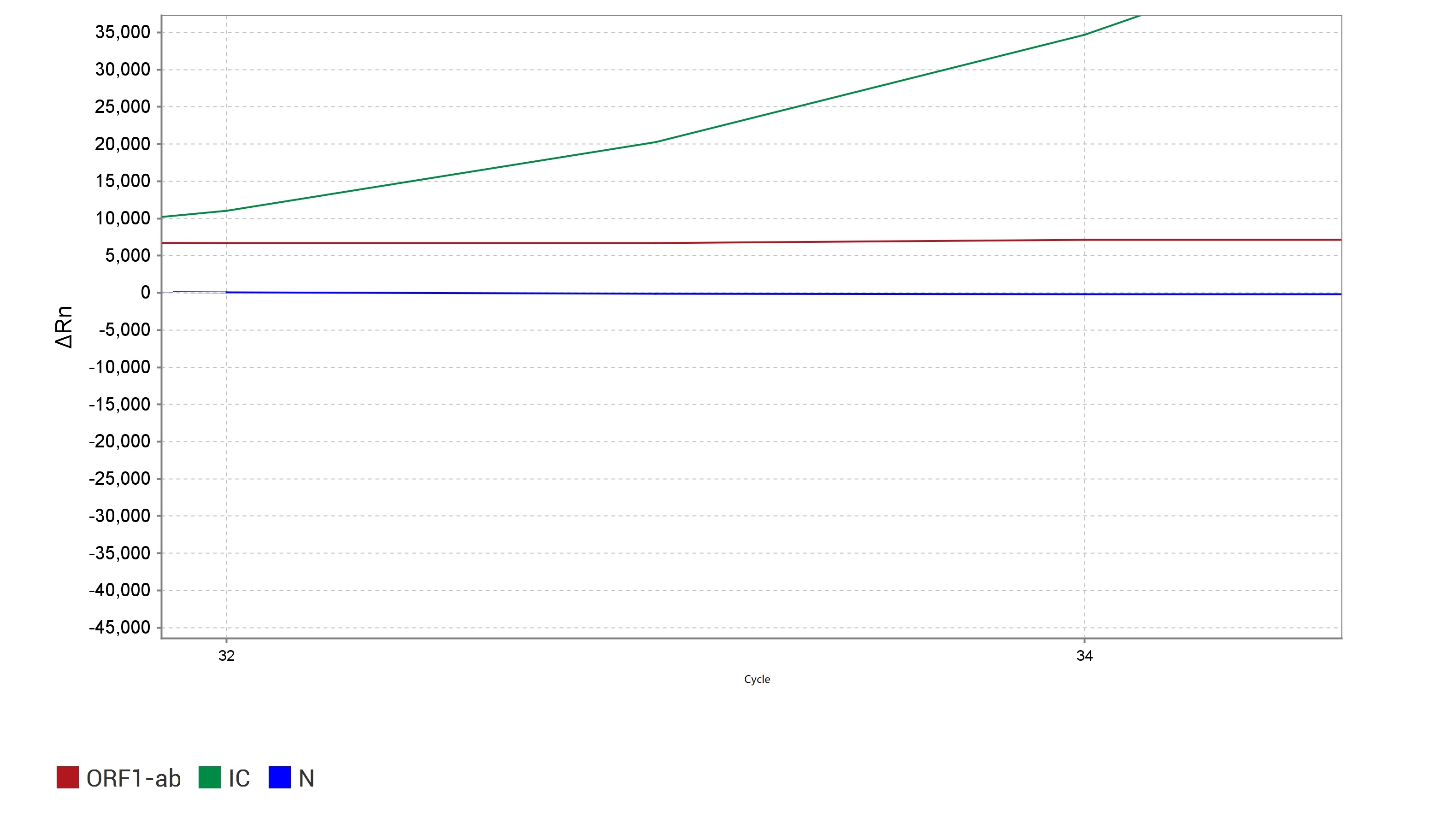

Supplement: S1 File — (ZIP) [file pone.0286121.s001.zip › DNA amplification graphs English/general ward Contaminated area Bed rai 33.6 34.6.jpg]

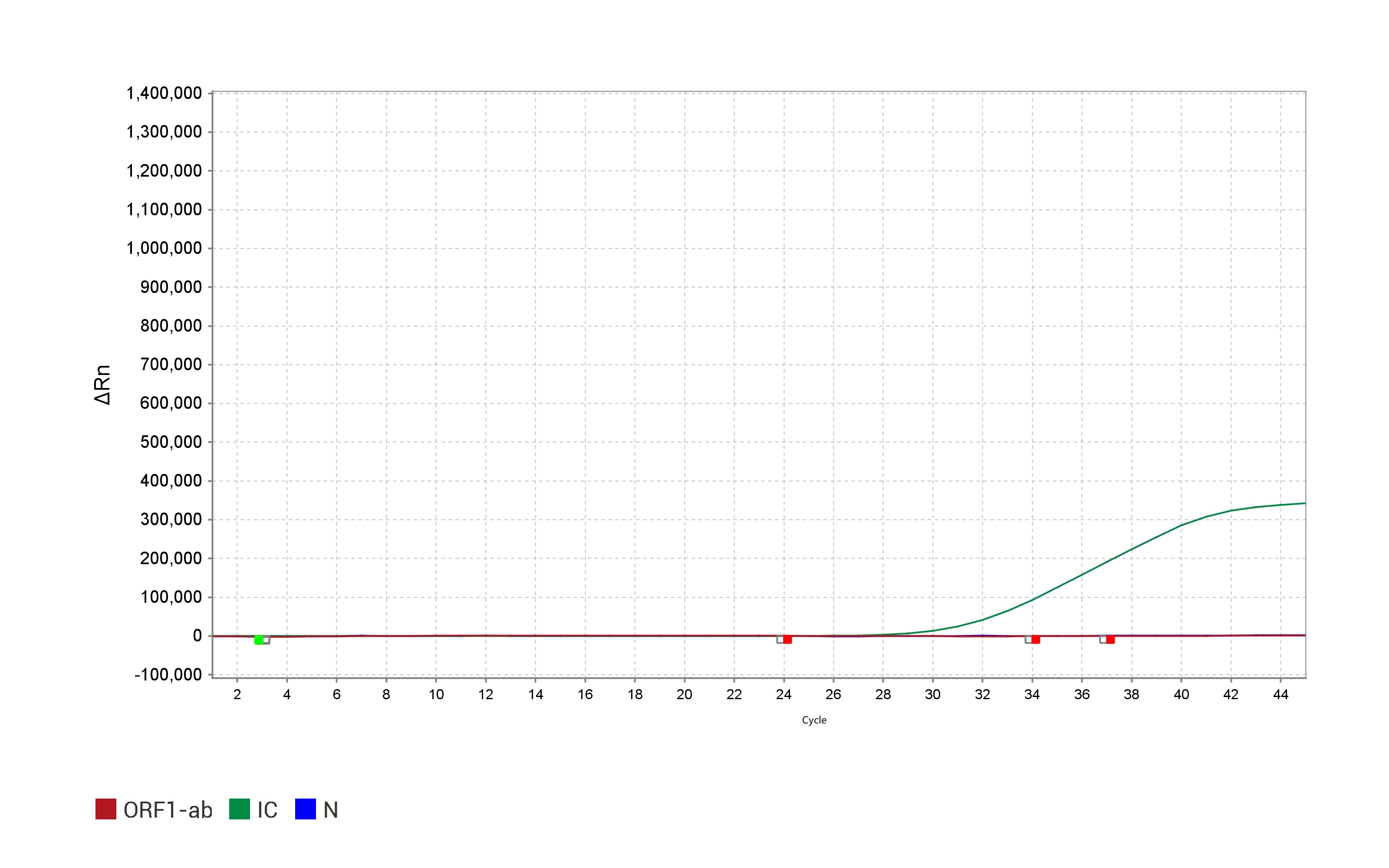

Supplement: S1 File — (ZIP) [file pone.0286121.s001.zip › DNA amplification graphs English/general ward Contaminated area Door handle 33.4 33.0.jpg]

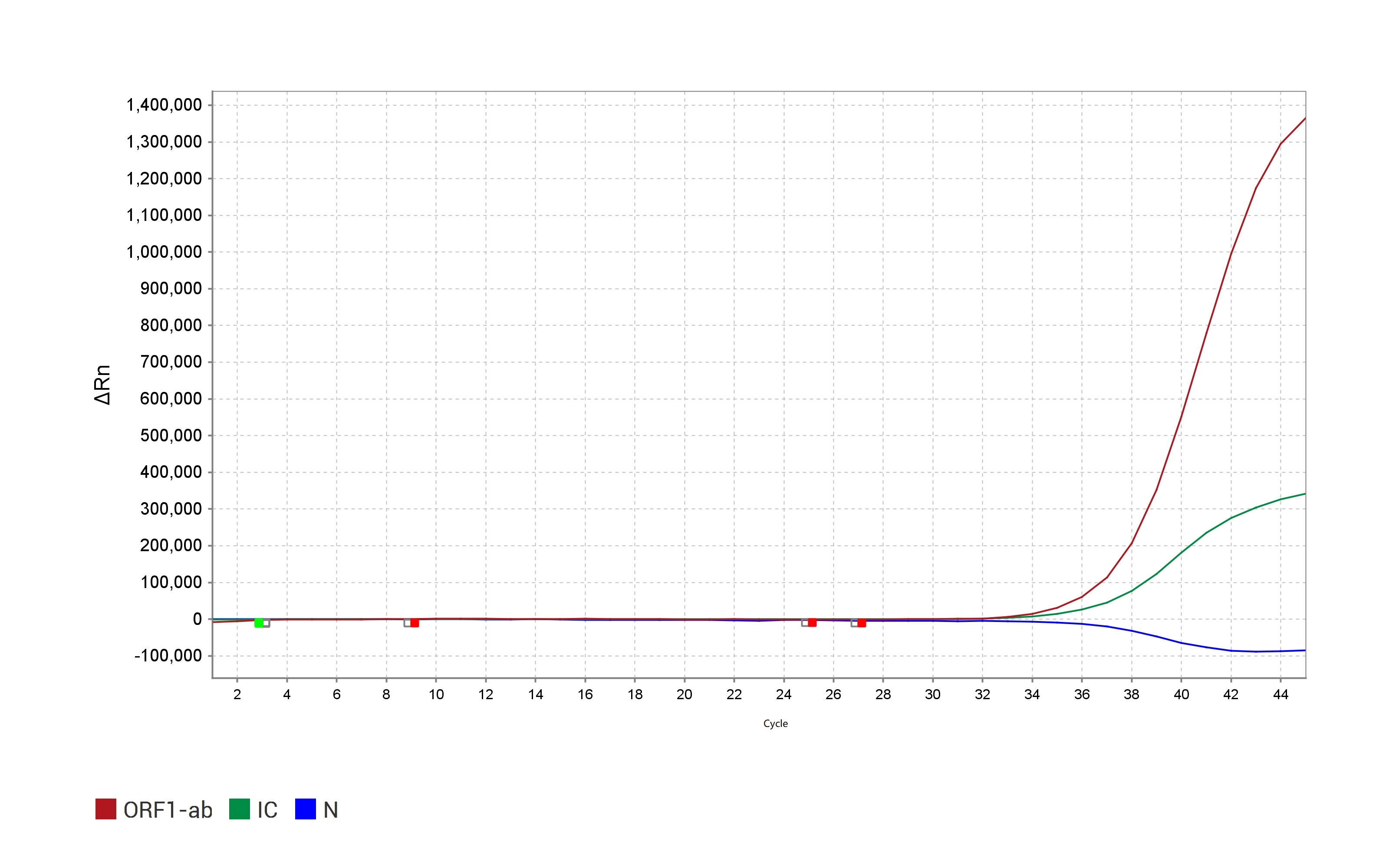

Supplement: S1 File — (ZIP) [file pone.0286121.s001.zip › DNA amplification graphs English/general ward Contaminated area Pillow 34.8.jpg]

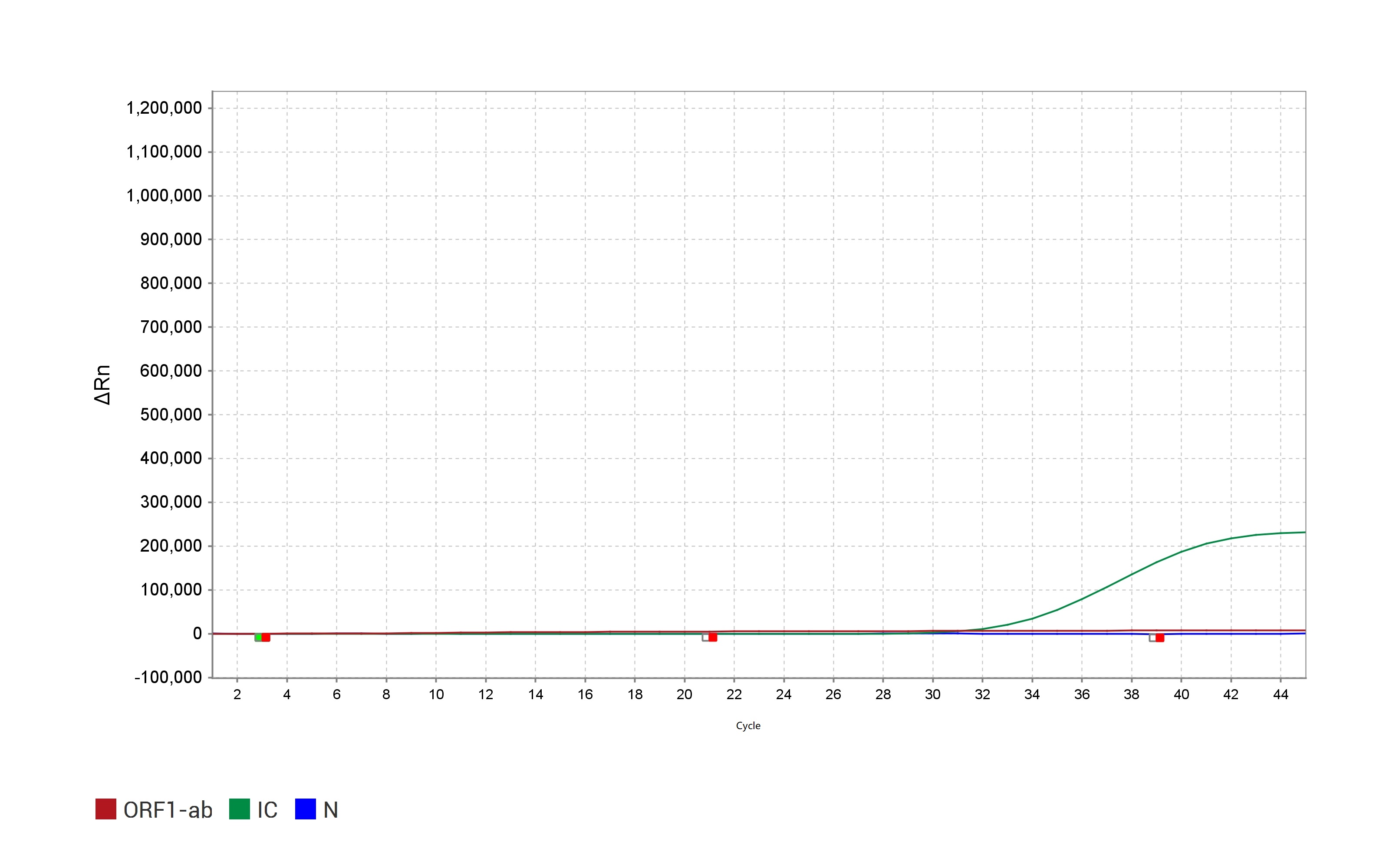

Supplement: S1 File — (ZIP) [file pone.0286121.s001.zip › DNA amplification graphs English/general ward Contaminated aread Personal protective equipment 34.0.jpg]

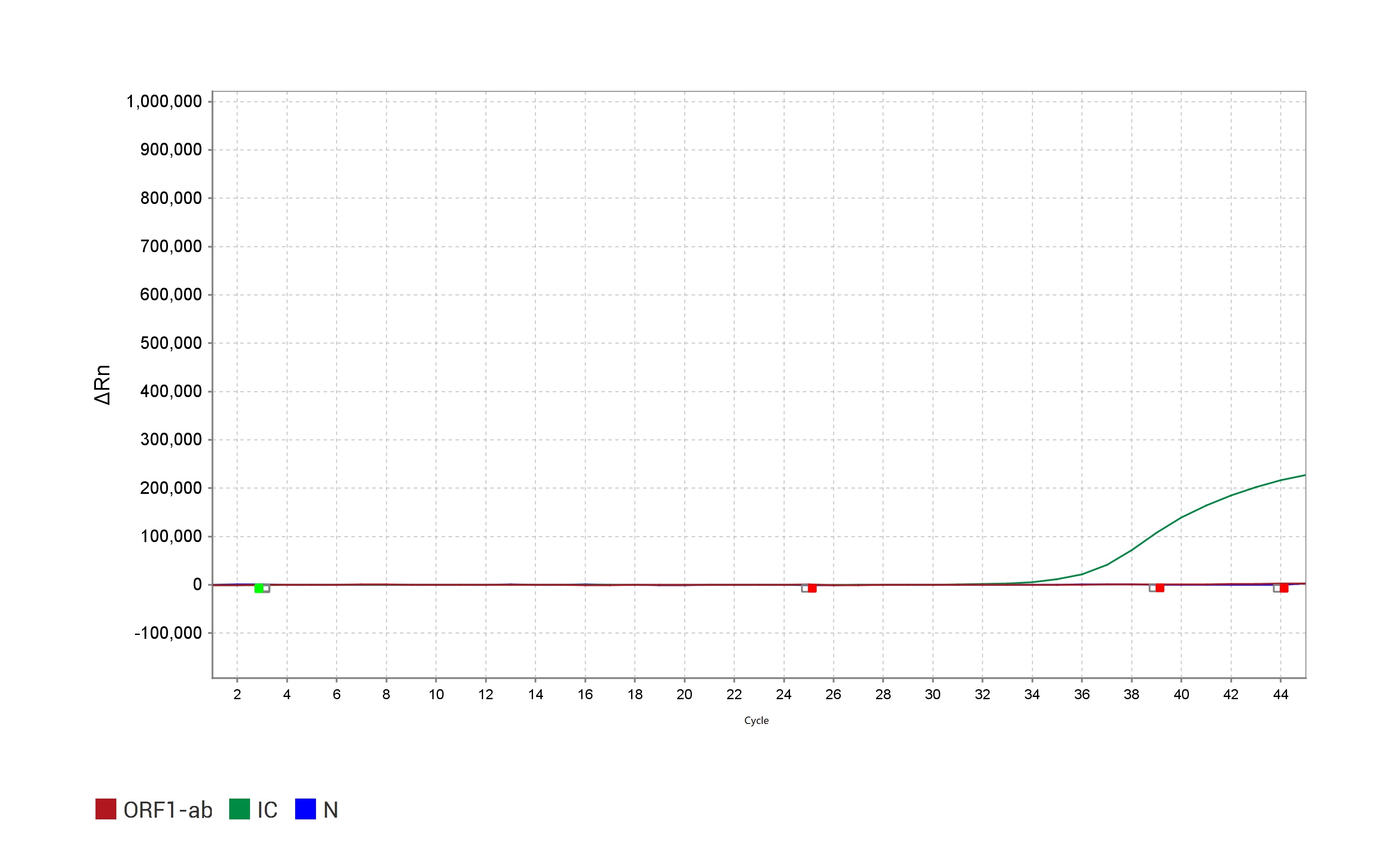

Supplement: S1 File — (ZIP) [file pone.0286121.s001.zip › DNA amplification graphs English/general ward Contaminated area Bed rail 39.0.jpg]

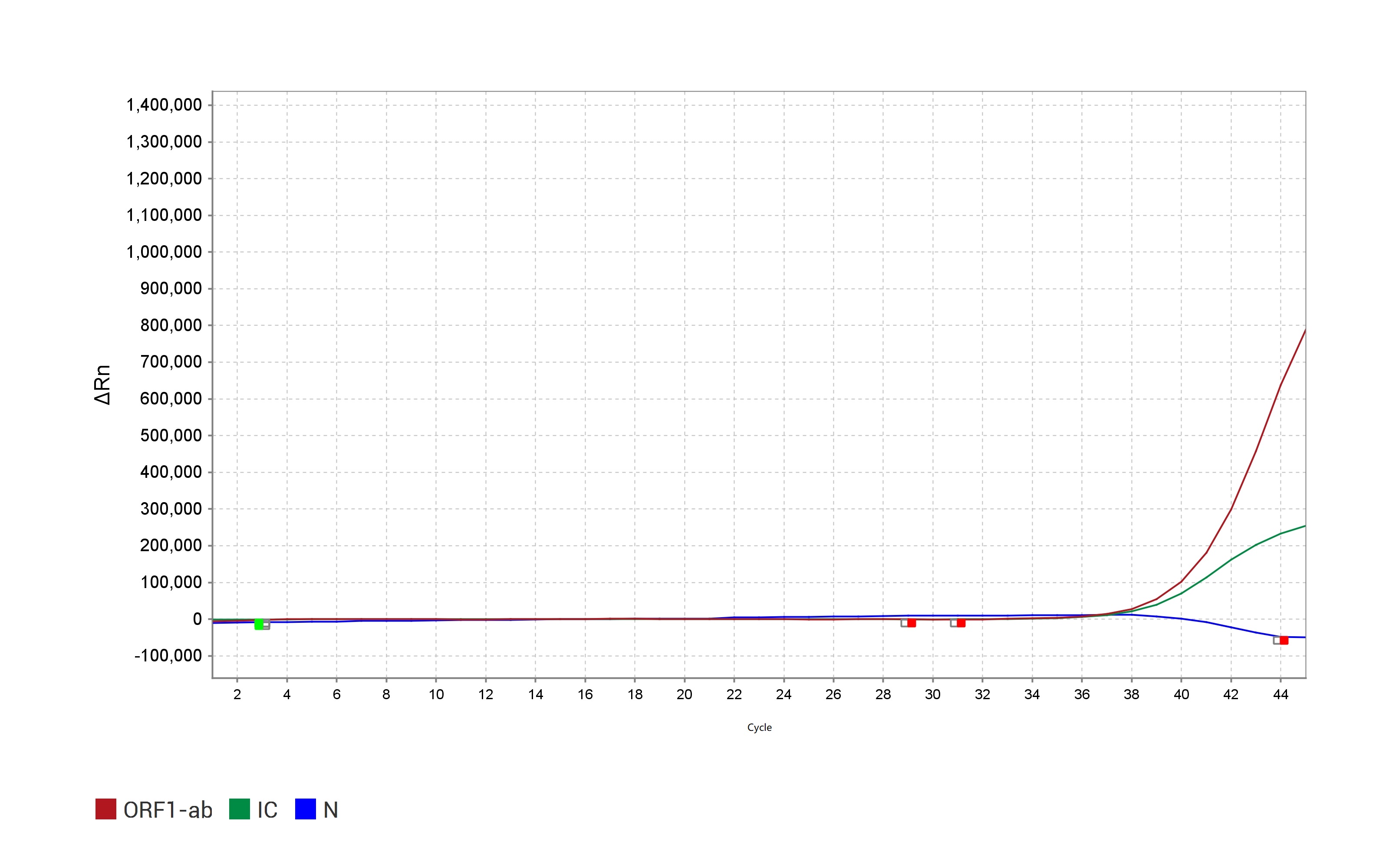

Supplement: S1 File — (ZIP) [file pone.0286121.s001.zip › DNA amplification graphs English/general ward Contaminated area Medical equipment 37.7.jpg]

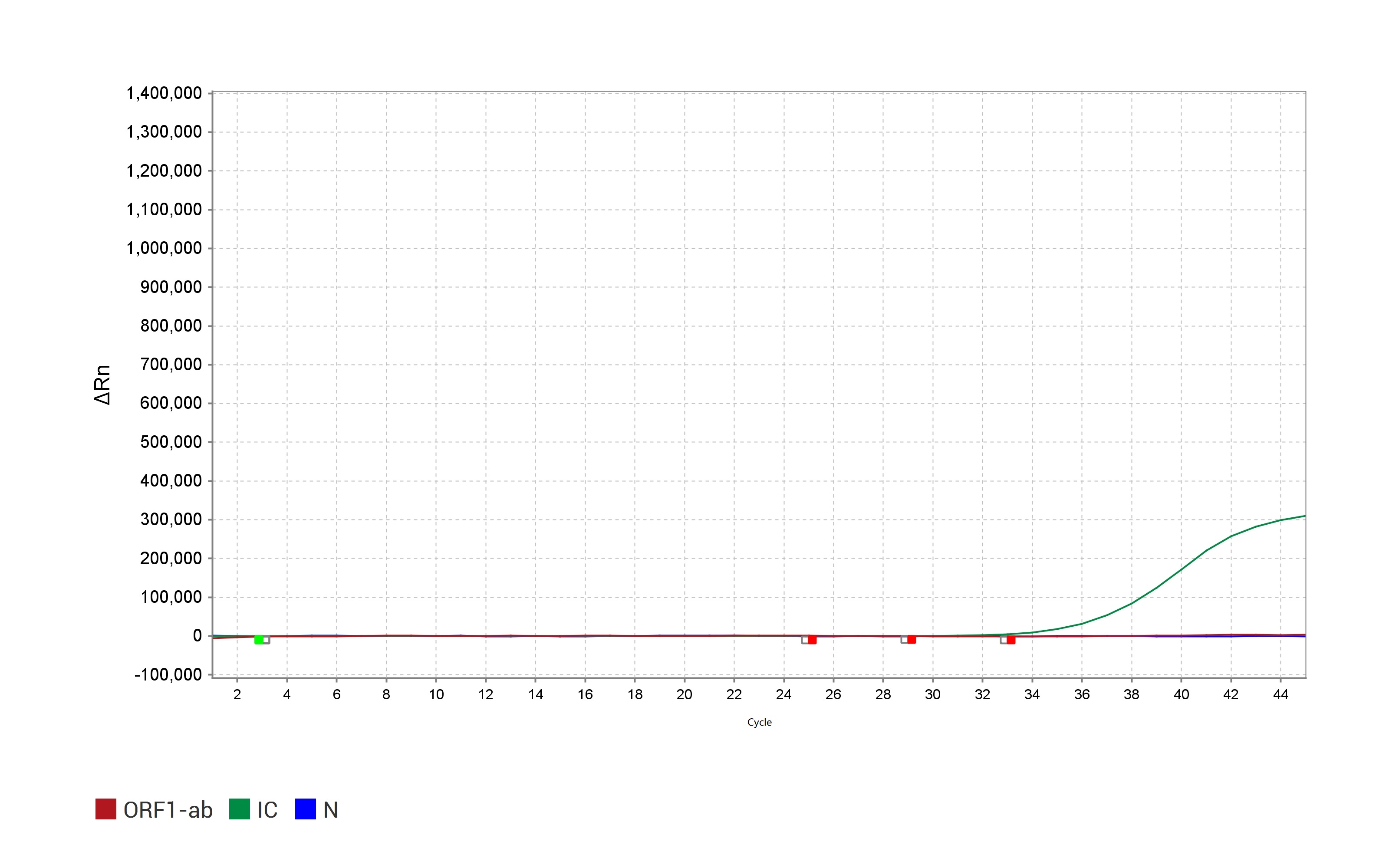

Supplement: S1 File — (ZIP) [file pone.0286121.s001.zip › DNA amplification graphs English/general ward Contaminated area Medical equipment 37.9 36.8.jpg]

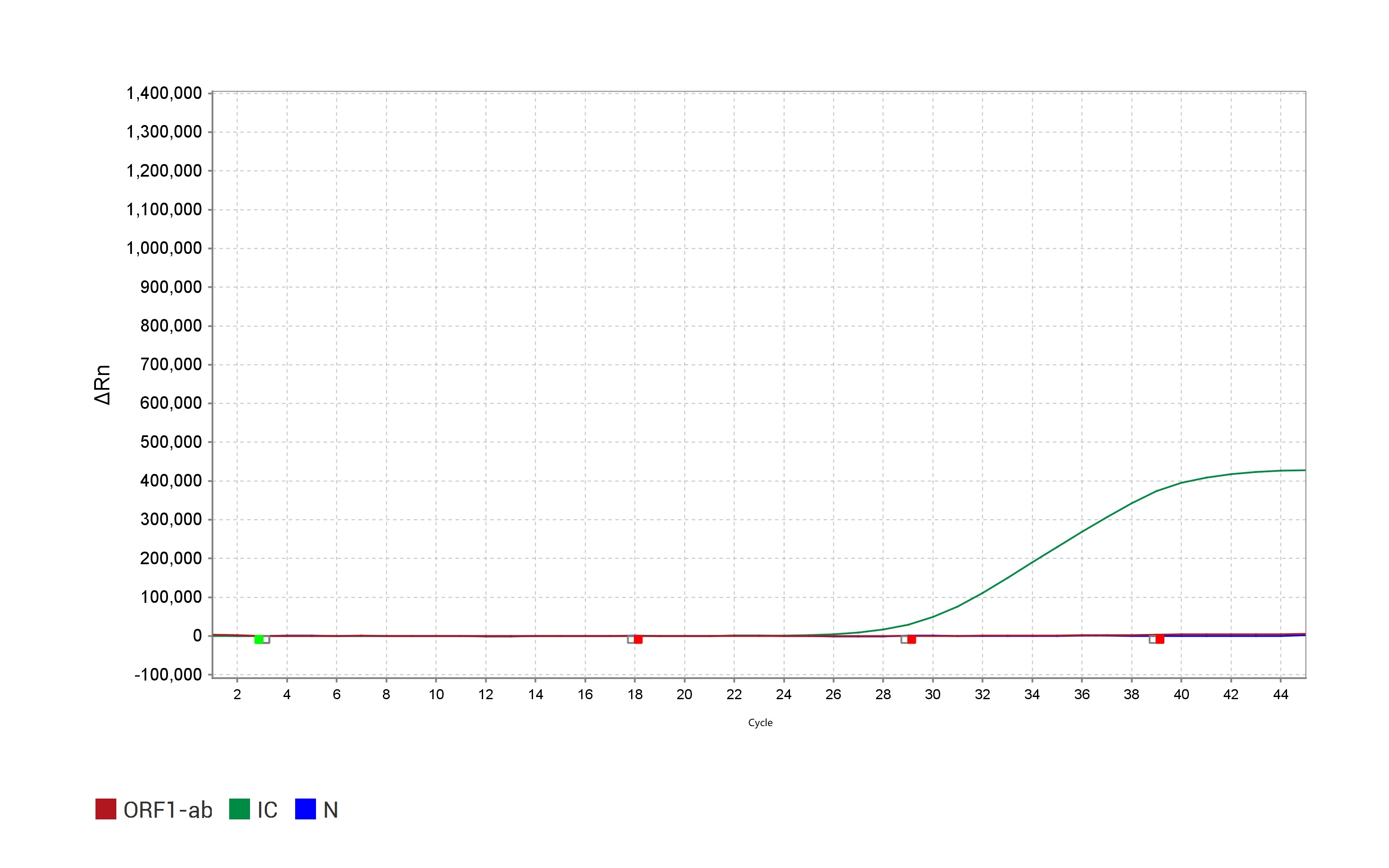

Supplement: S1 File — (ZIP) [file pone.0286121.s001.zip › DNA amplification graphs English/general ward Contaminated area Bed rail 38.6 38.6.jpg]

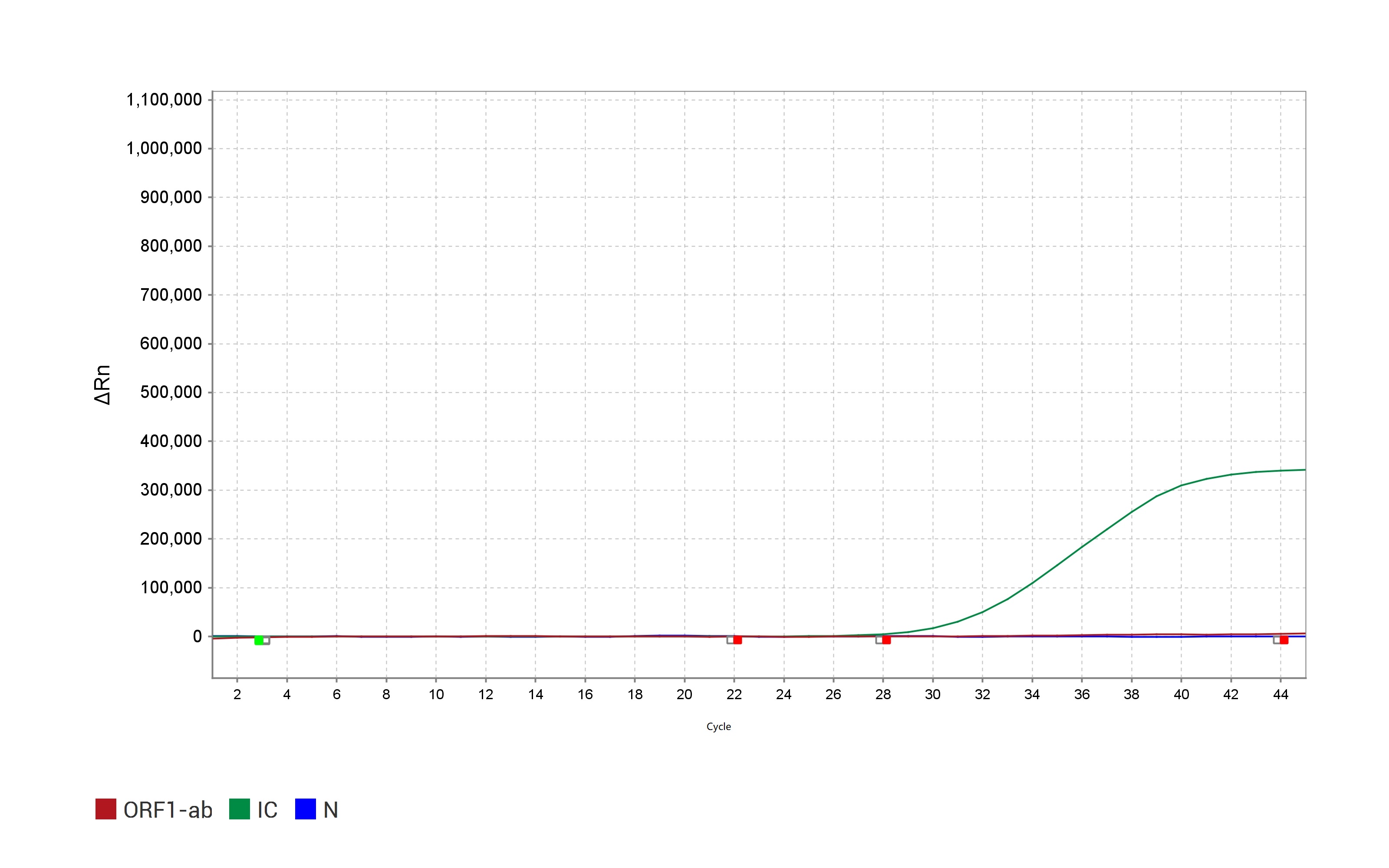

Supplement: S1 File — (ZIP) [file pone.0286121.s001.zip › DNA amplification graphs English/general ward Contaminated area Bed rail 33.9.jpg]

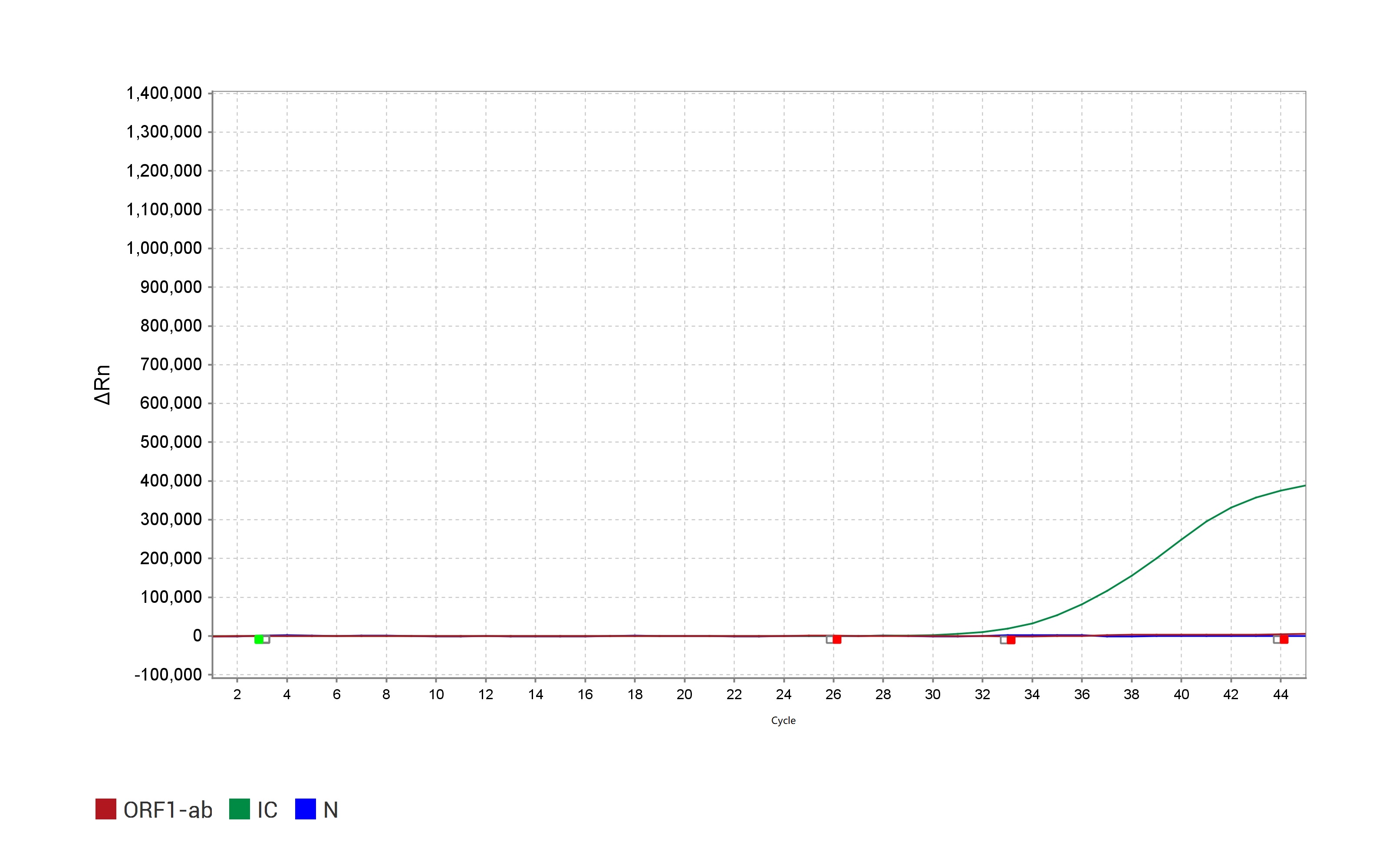

Supplement: S1 File — (ZIP) [file pone.0286121.s001.zip › DNA amplification graphs English/general ward Contaminated area Bed rail 35.5.jpg]

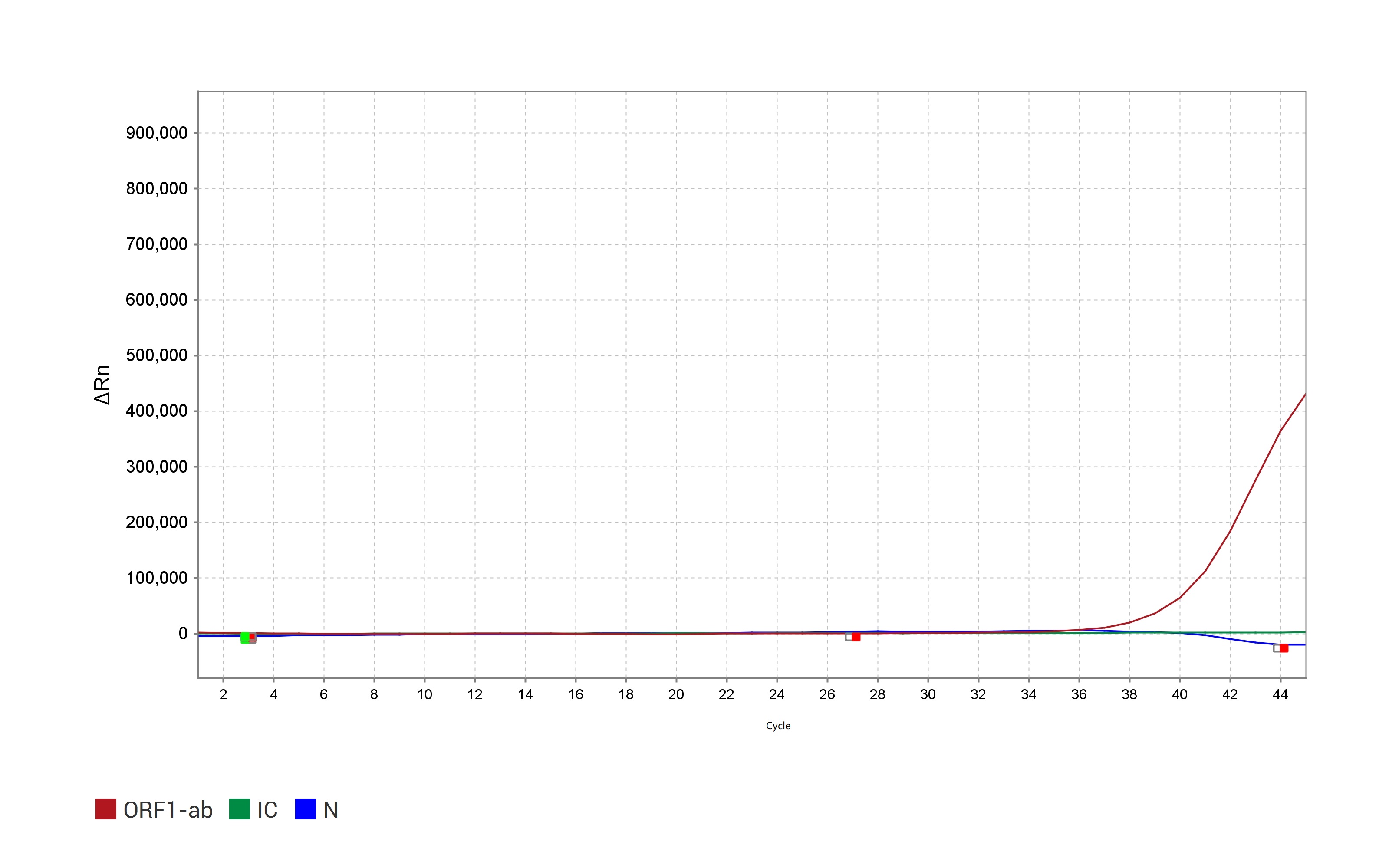

Supplement: S1 File — (ZIP) [file pone.0286121.s001.zip › DNA amplification graphs English/general ward Contaminated area Bed rail 37.6.jpg]

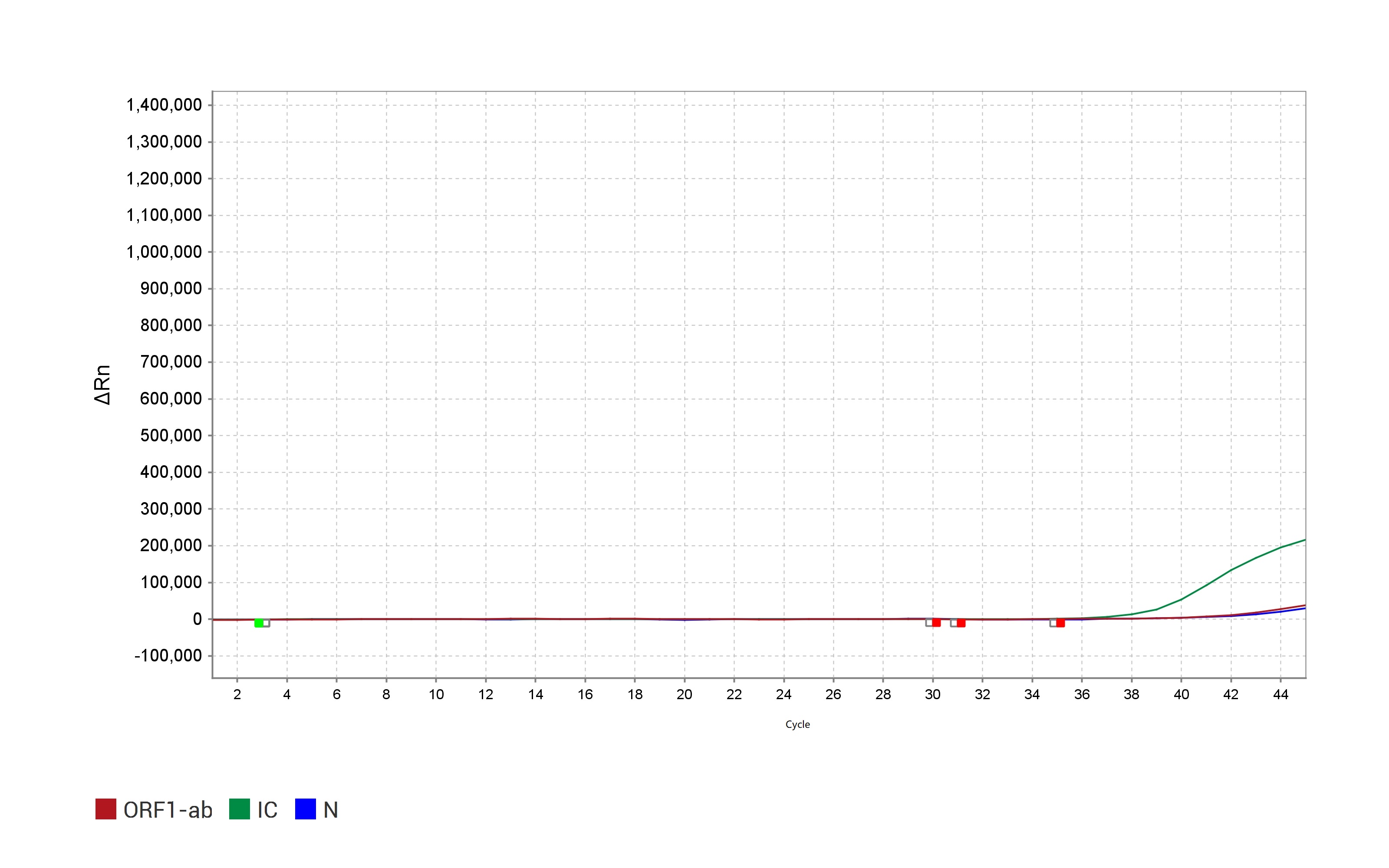

Supplement: S1 File — (ZIP) [file pone.0286121.s001.zip › DNA amplification graphs English/general ward Contaminated area Bed stand 38.7 37.2.jpg]

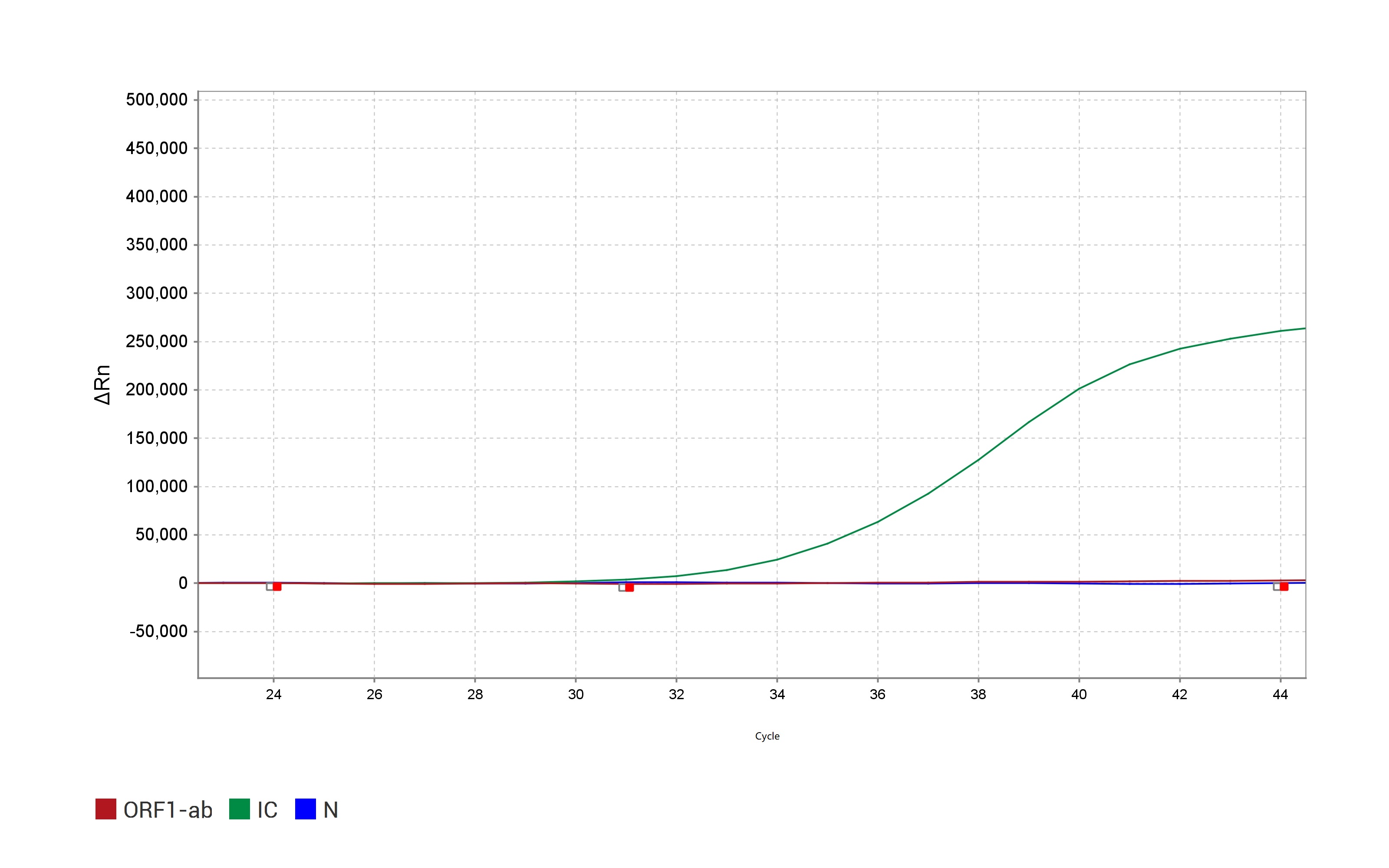

Supplement: S1 File — (ZIP) [file pone.0286121.s001.zip › DNA amplification graphs English/general ward Contaminated area Bed stand 37.0 37.7.jpg]

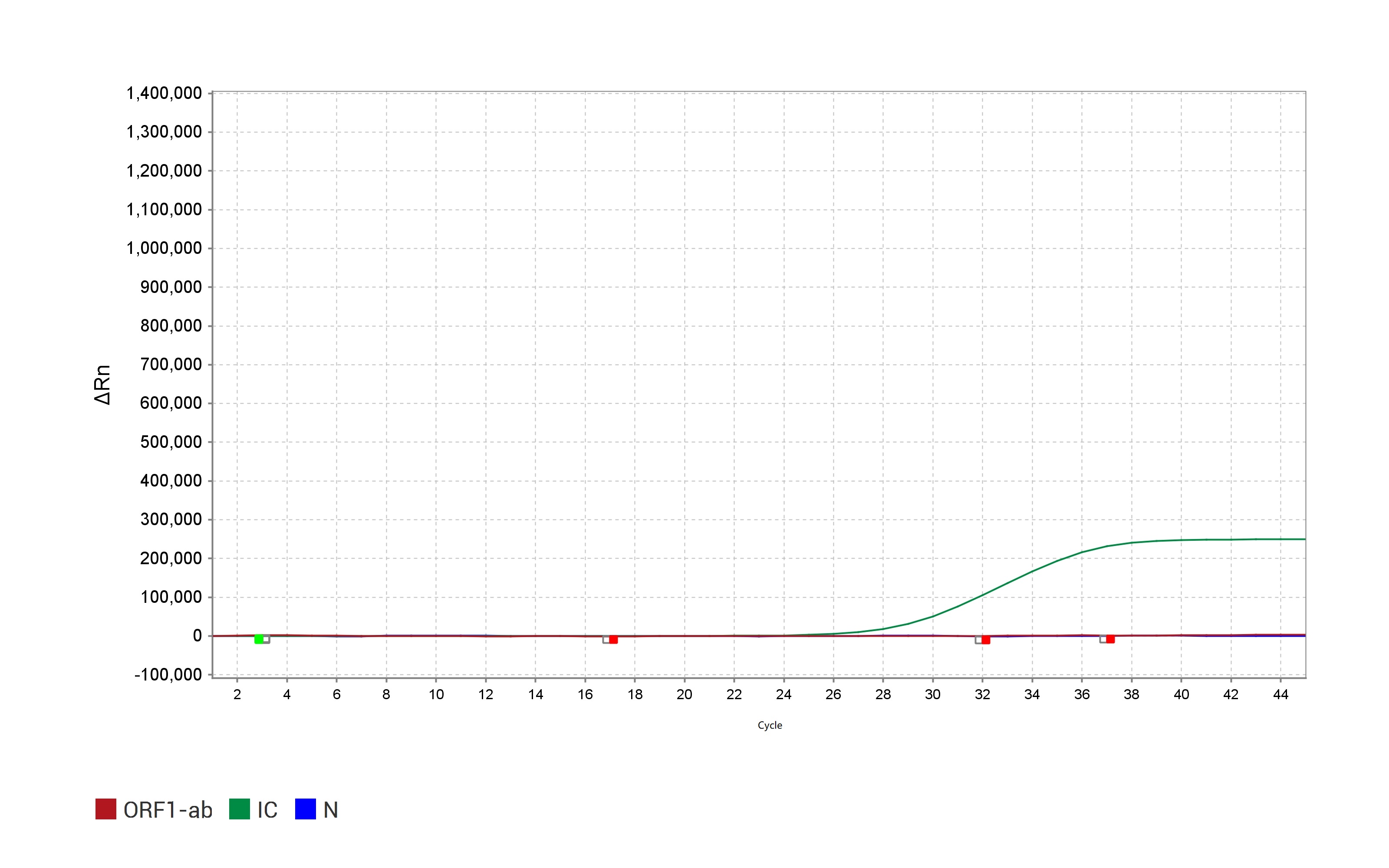

Supplement: S1 File — (ZIP) [file pone.0286121.s001.zip › DNA amplification graphs English/general ward Contaminated area Door handle 37.3 37.9.jpg]

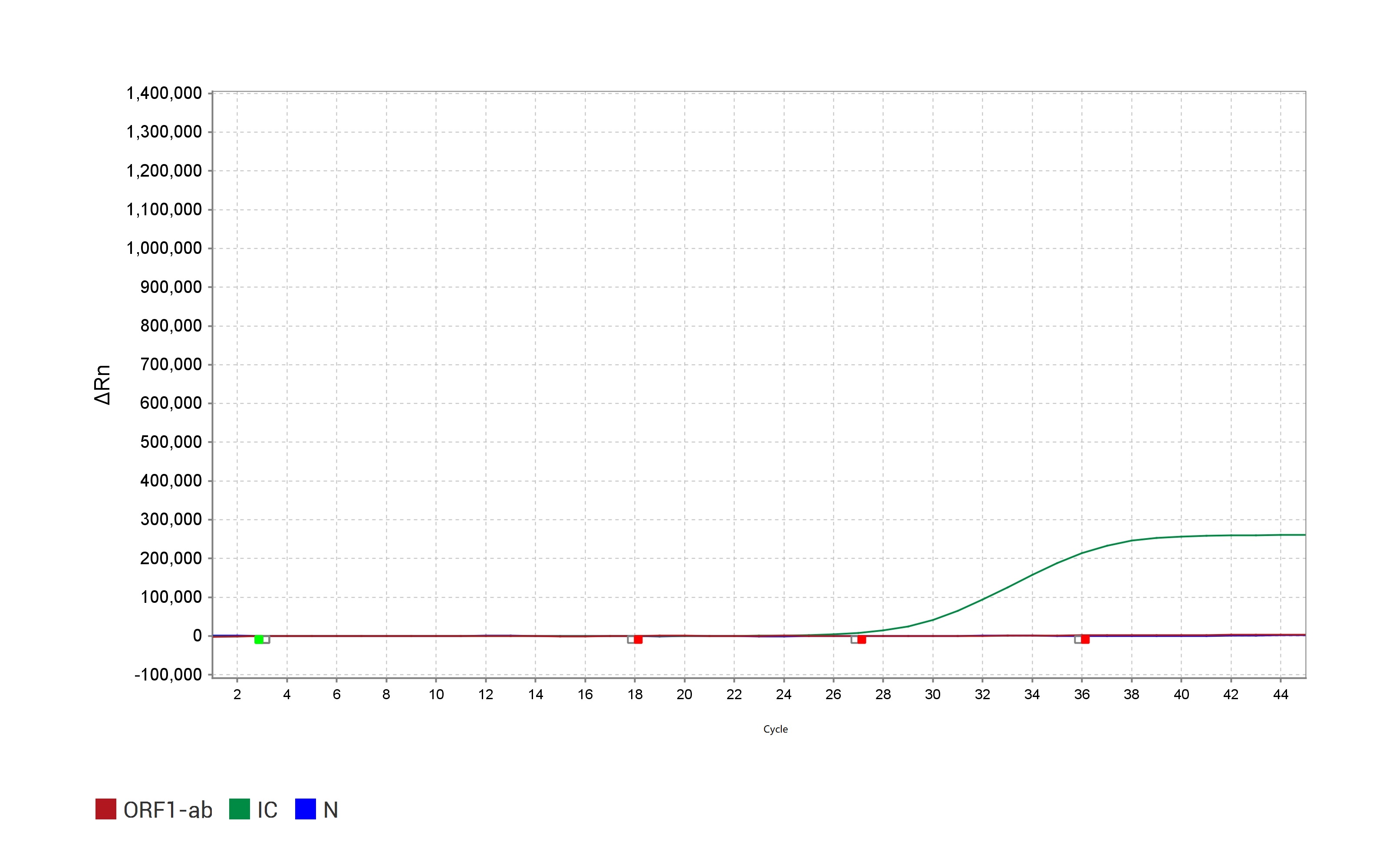

Supplement: S1 File — (ZIP) [file pone.0286121.s001.zip › DNA amplification graphs English/general ward Contaminated area Equipment belt 34.8 36.5.jpg]

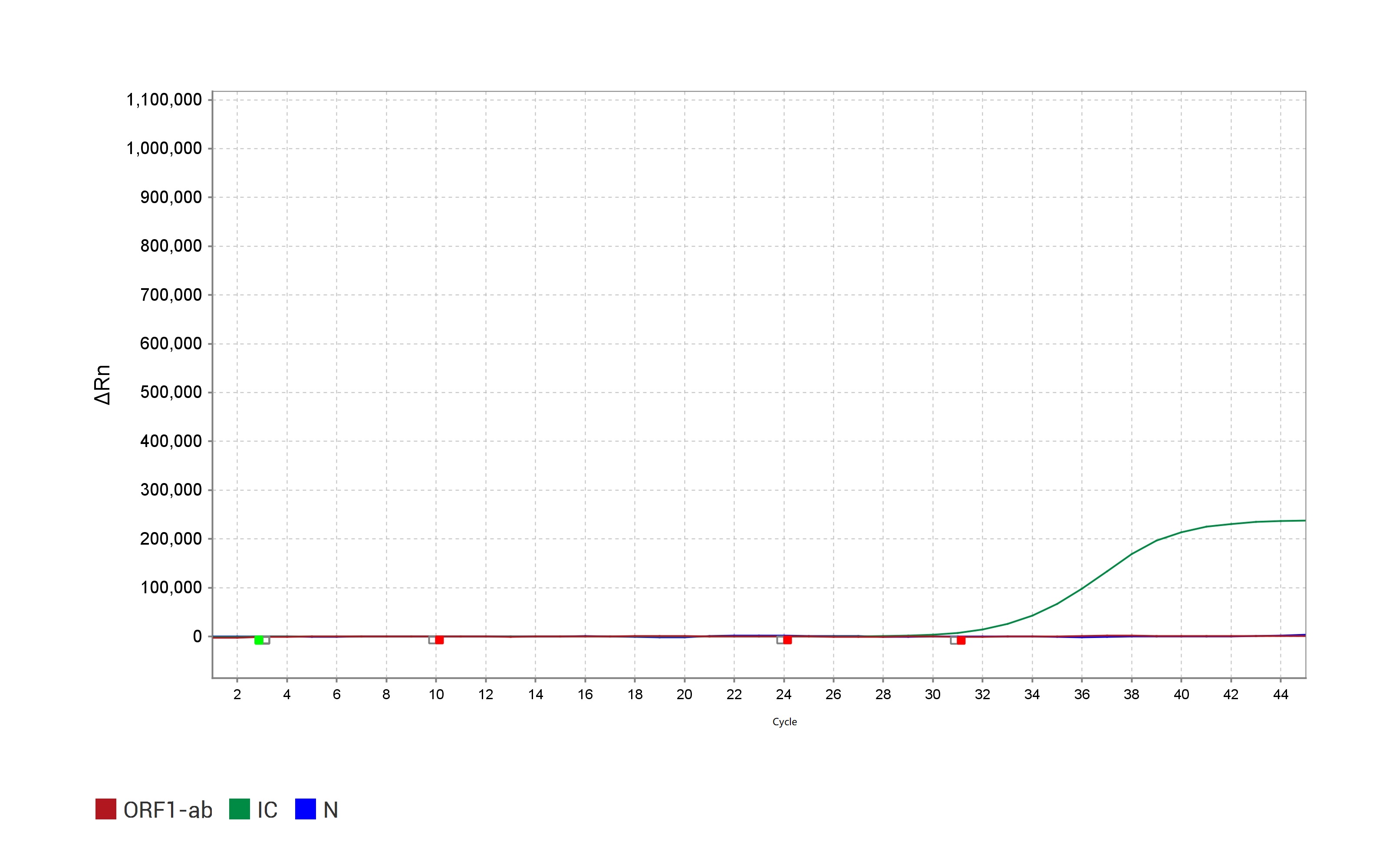

Supplement: S1 File — (ZIP) [file pone.0286121.s001.zip › DNA amplification graphs English/general ward Contaminated area Equipment belt 37.2 37.6 .jpg]

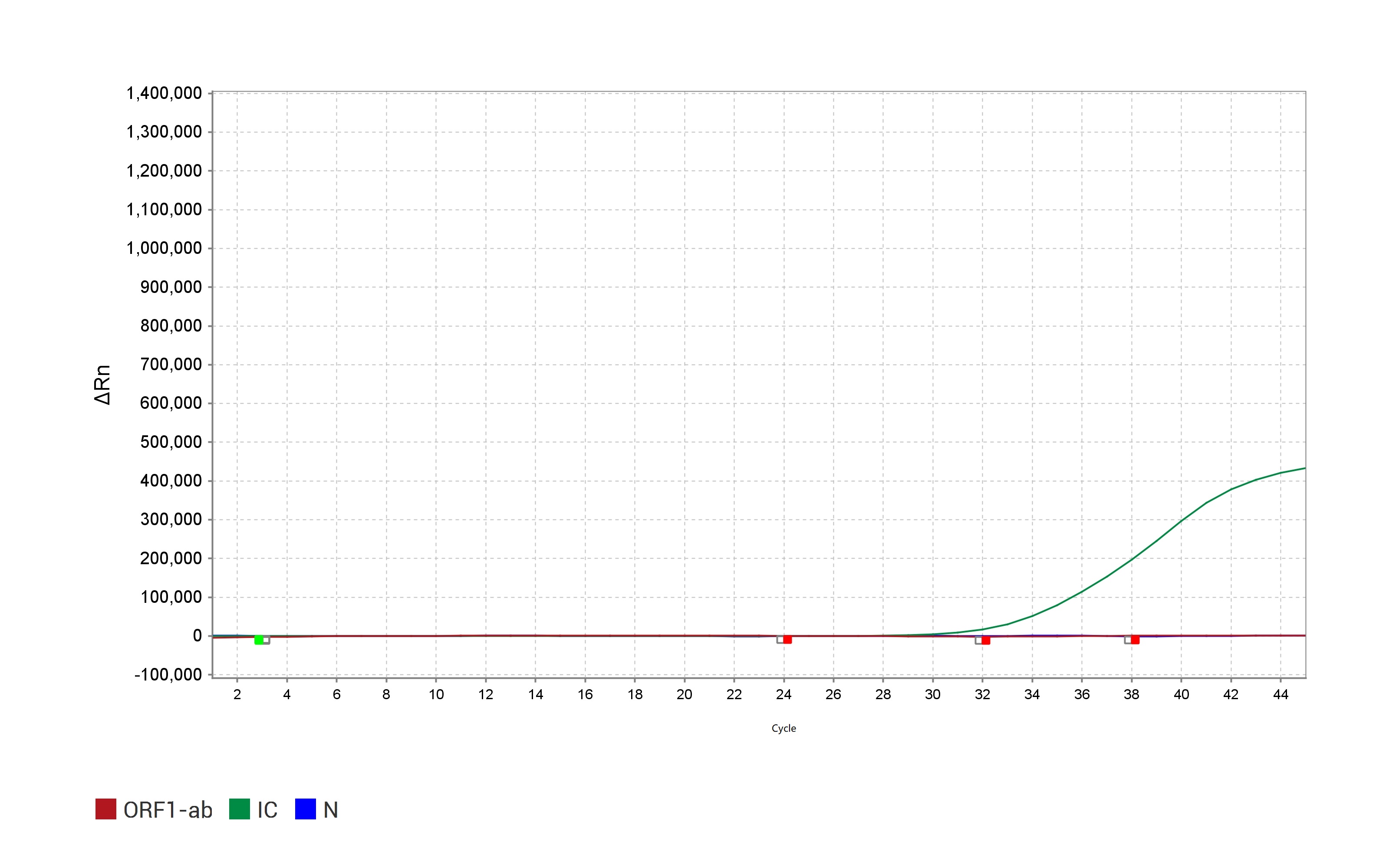

Supplement: S1 File — (ZIP) [file pone.0286121.s001.zip › DNA amplification graphs English/general ward Contaminated area Equipment belt35.5 37.3.jpg]

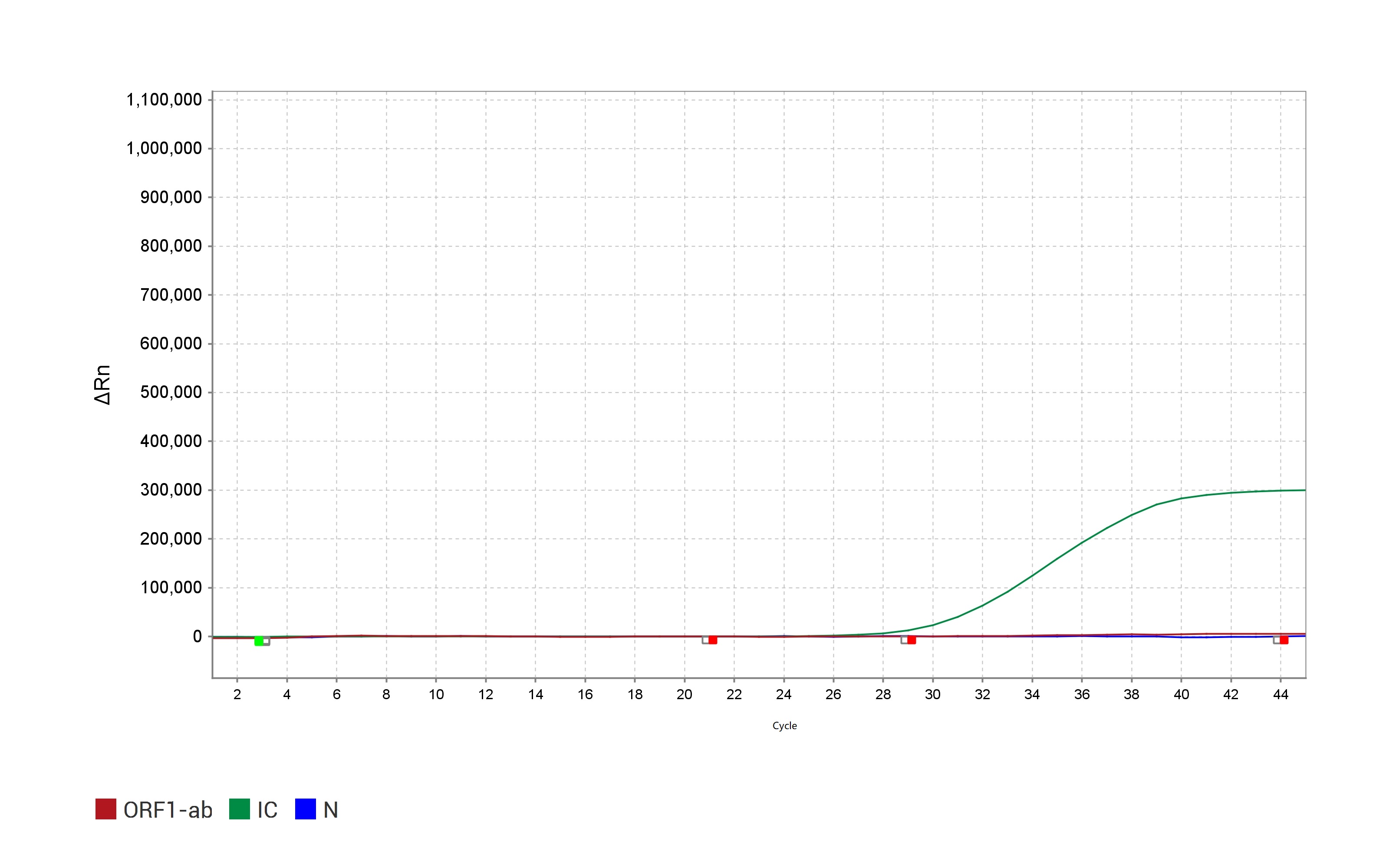

Supplement: S1 File — (ZIP) [file pone.0286121.s001.zip › DNA amplification graphs English/general ward Contaminated area Infusion stand 33.6.jpg]

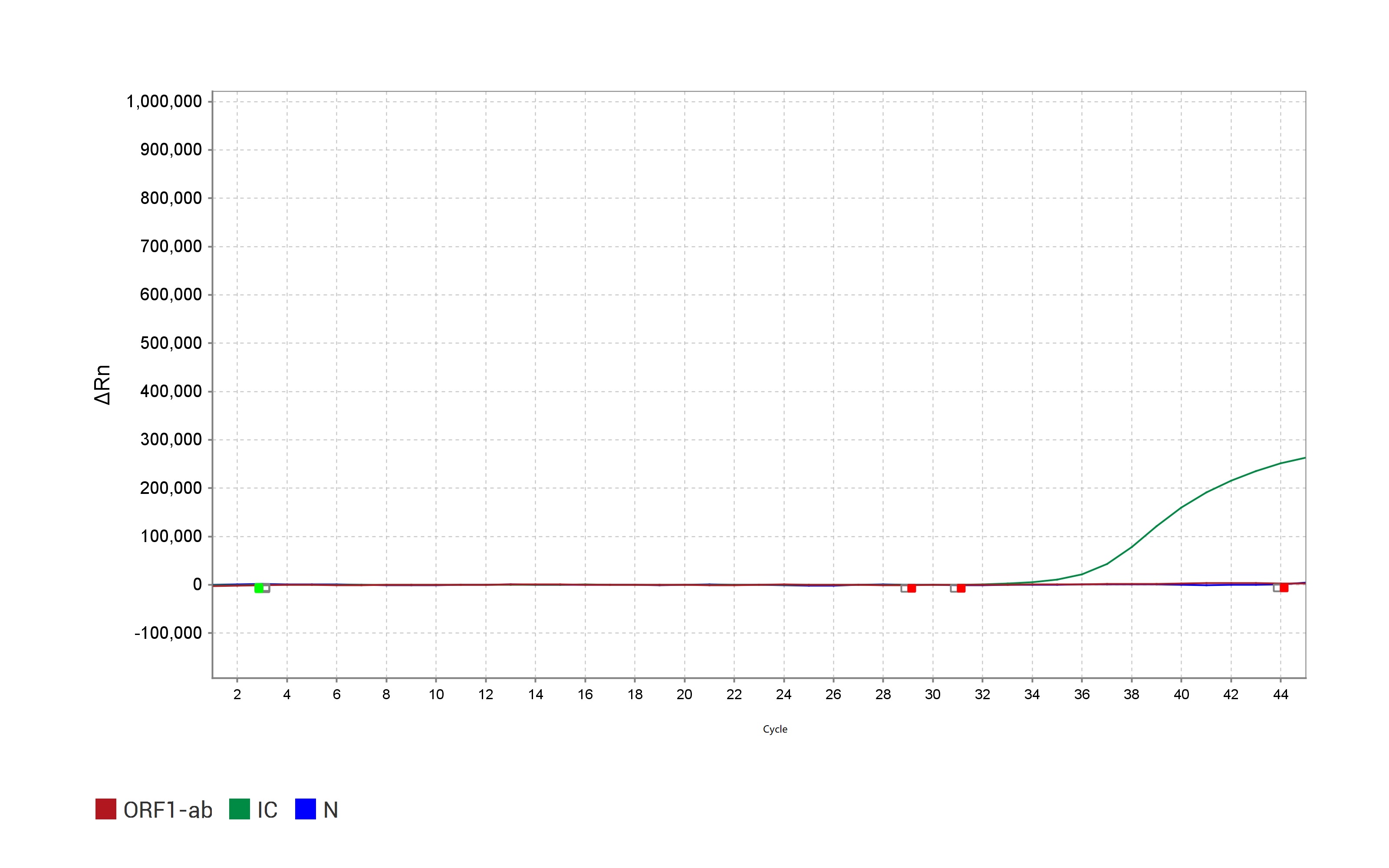

Supplement: S1 File — (ZIP) [file pone.0286121.s001.zip › DNA amplification graphs English/general ward Contaminated area Medical equipment 34.6.jpg]

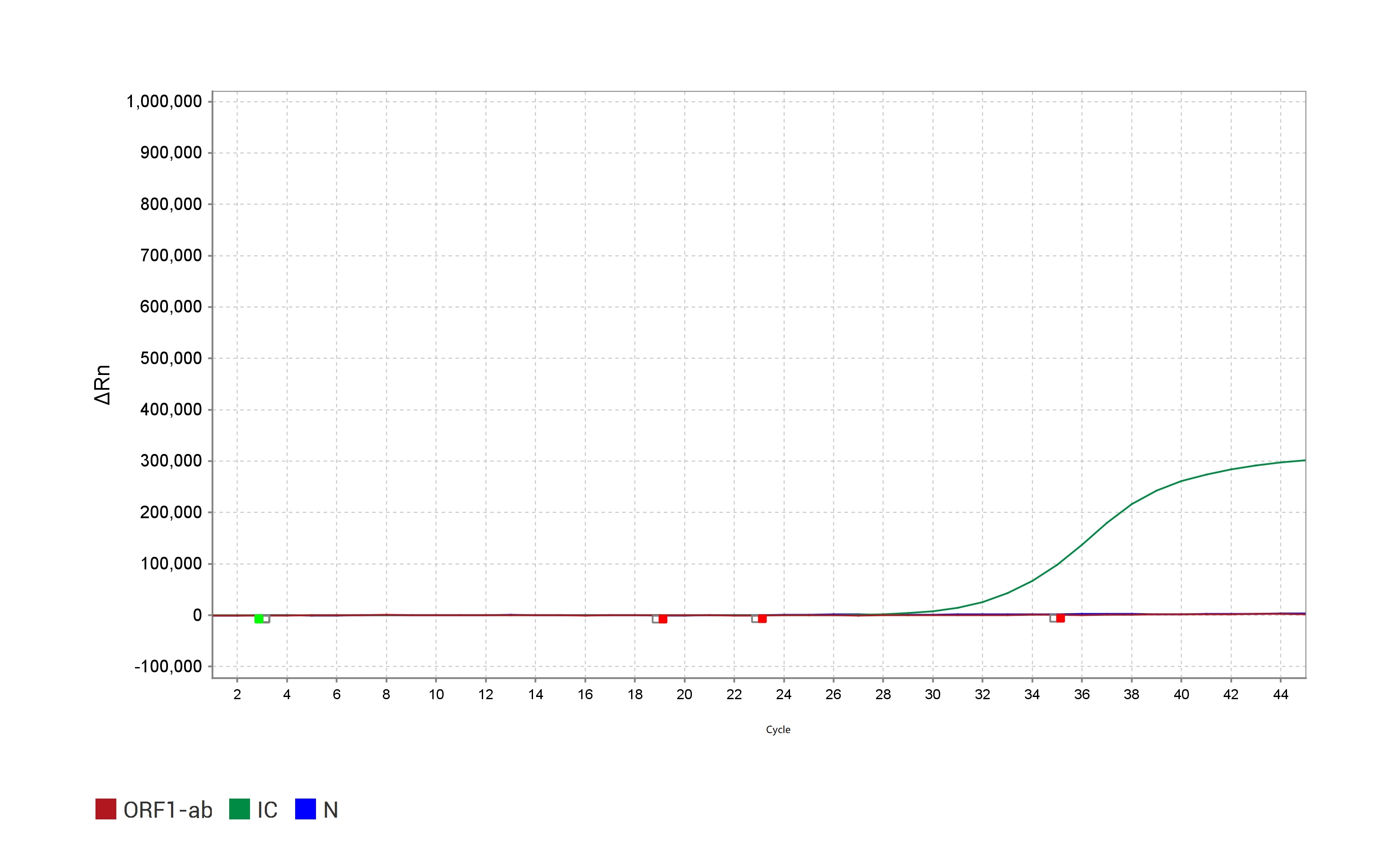

Supplement: S1 File — (ZIP) [file pone.0286121.s001.zip › DNA amplification graphs English/general ward Contaminated area Medical equipment 37.9.jpg]

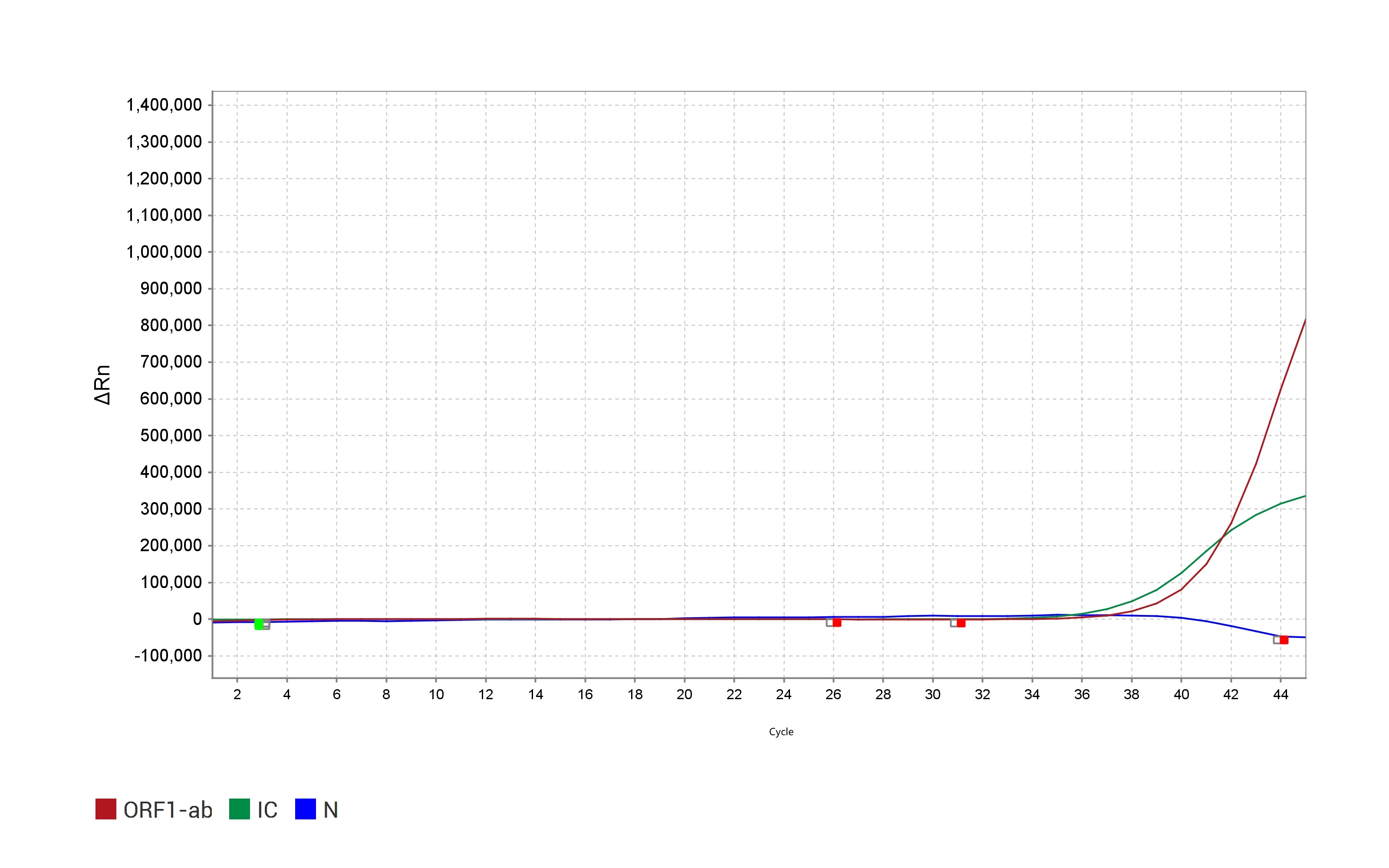

Supplement: S1 File — (ZIP) [file pone.0286121.s001.zip › DNA amplification graphs English/general ward Contaminated area Medical equipment 38.3.jpg]

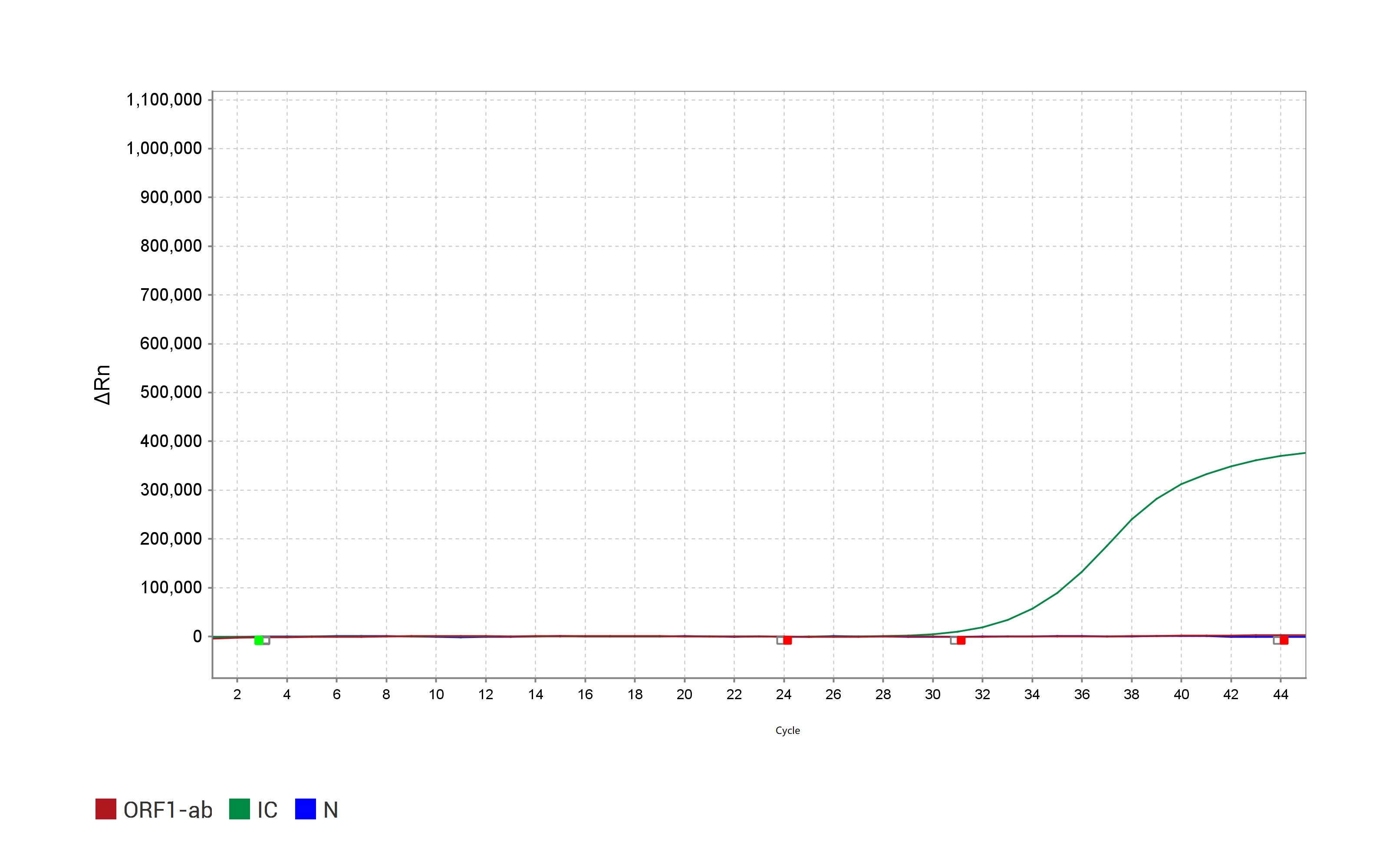

Supplement: S1 File — (ZIP) [file pone.0286121.s001.zip › DNA amplification graphs English/general ward Contaminated area Medical equipment 39.6.jpg]

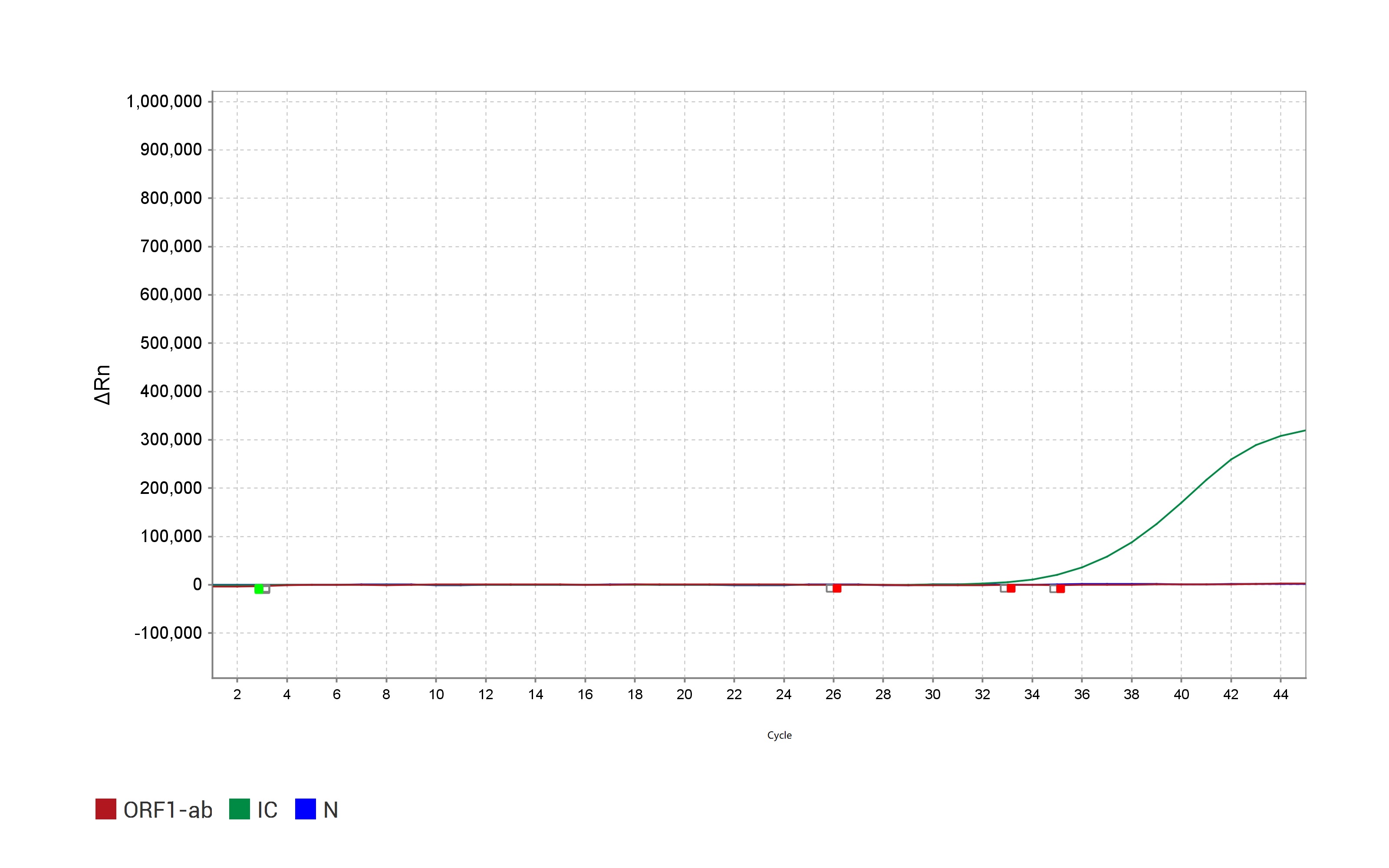

Supplement: S1 File — (ZIP) [file pone.0286121.s001.zip › DNA amplification graphs English/general ward Contaminated area Mobile phone 38.4 36.0.jpg]

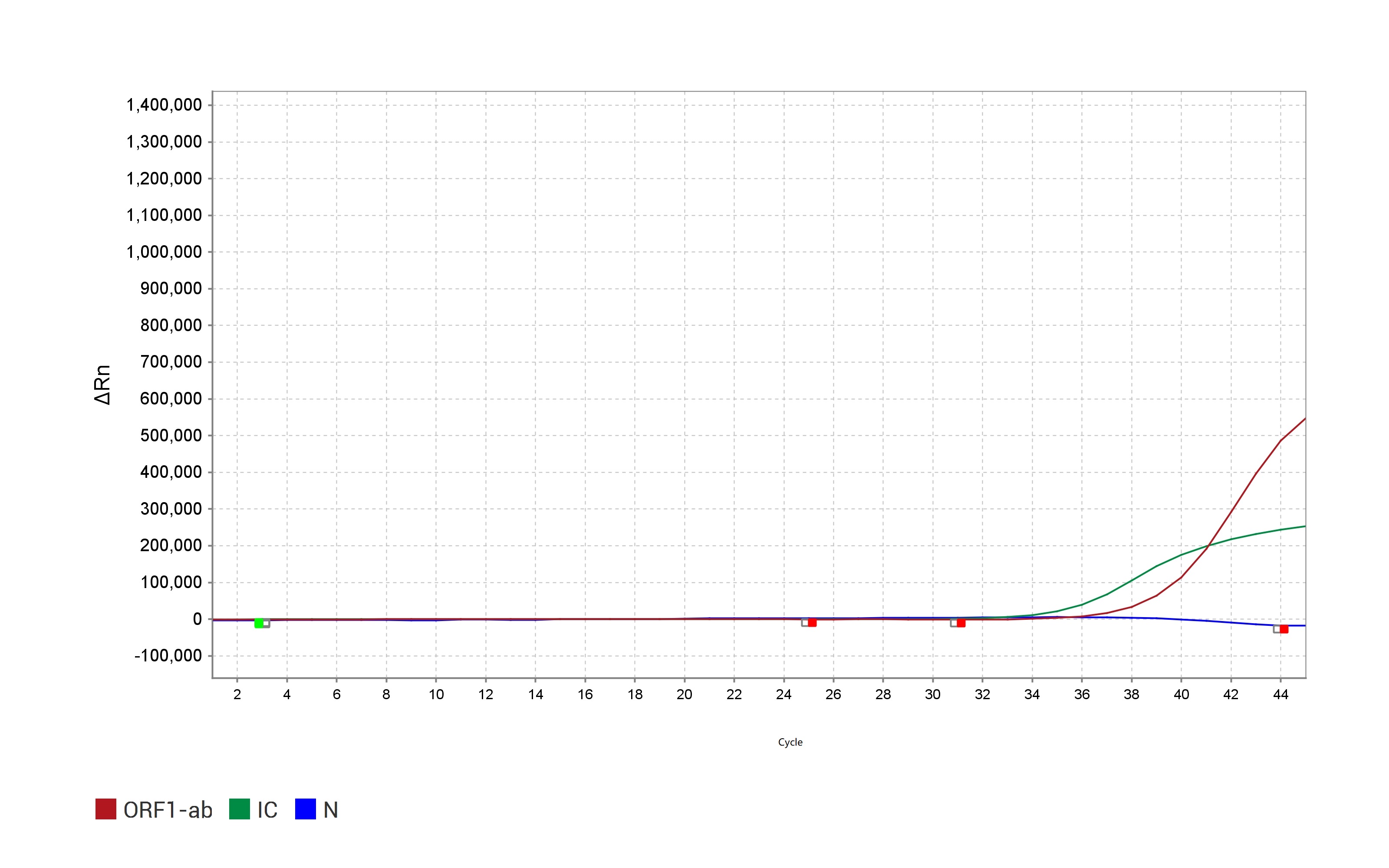

Supplement: S1 File — (ZIP) [file pone.0286121.s001.zip › DNA amplification graphs English/general ward Contaminated area Personal protective equipment 37.6.jpg]

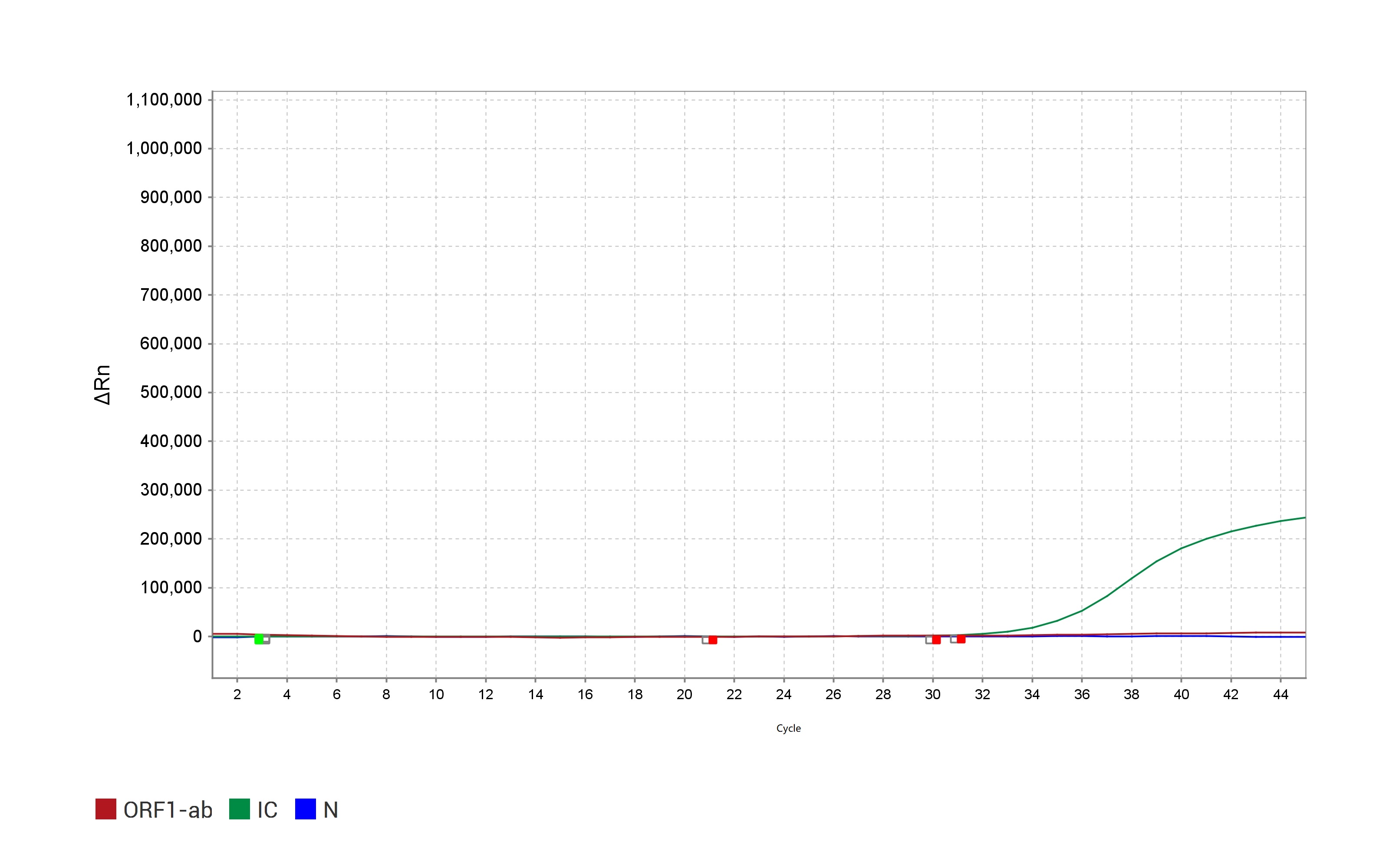

Supplement: S1 File — (ZIP) [file pone.0286121.s001.zip › DNA amplification graphs English/general ward Contaminated area Personal protective 32.2.jpg]

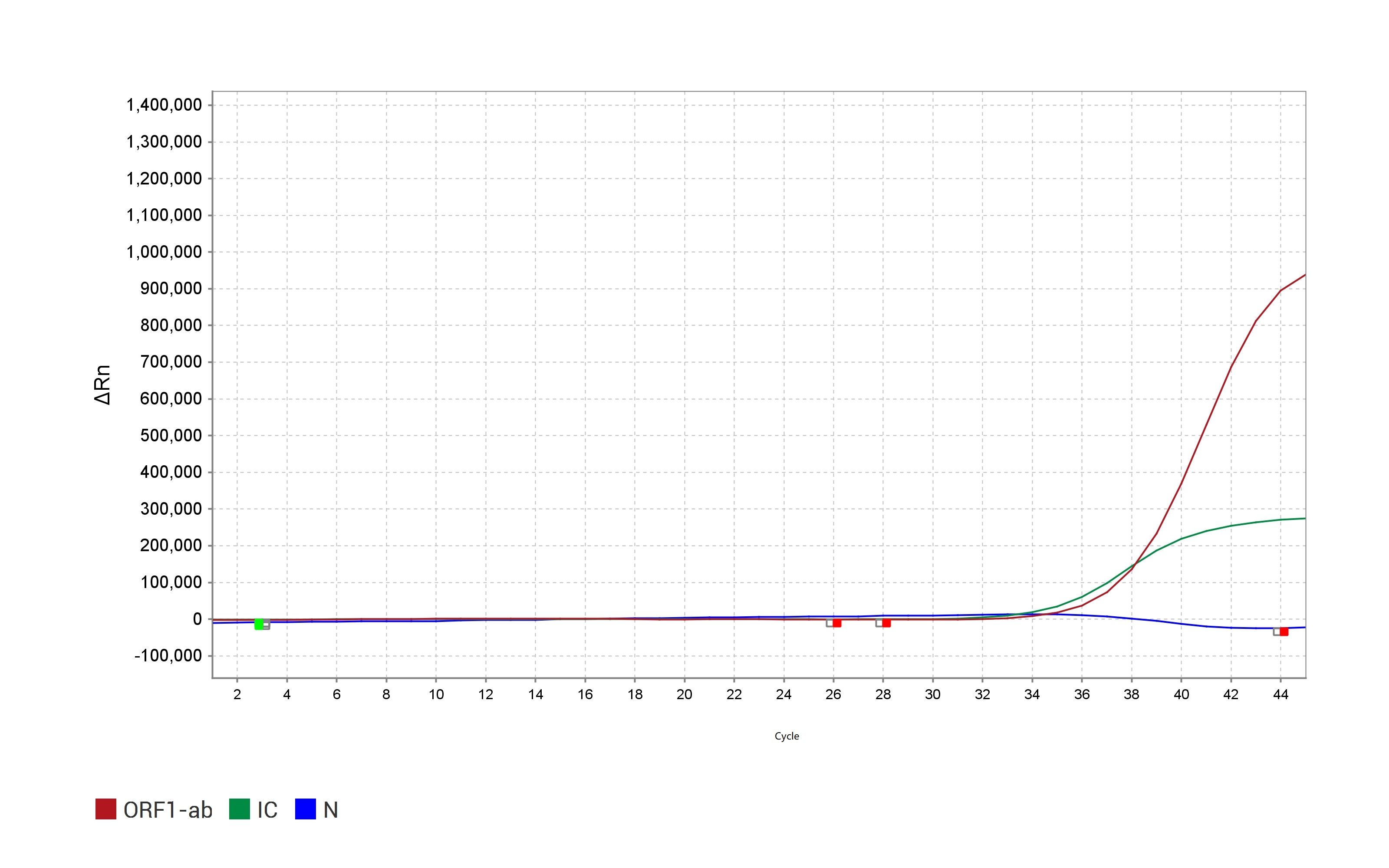

Supplement: S1 File — (ZIP) [file pone.0286121.s001.zip › DNA amplification graphs English/general ward Contaminated area Personal protective 35.5.jpg]

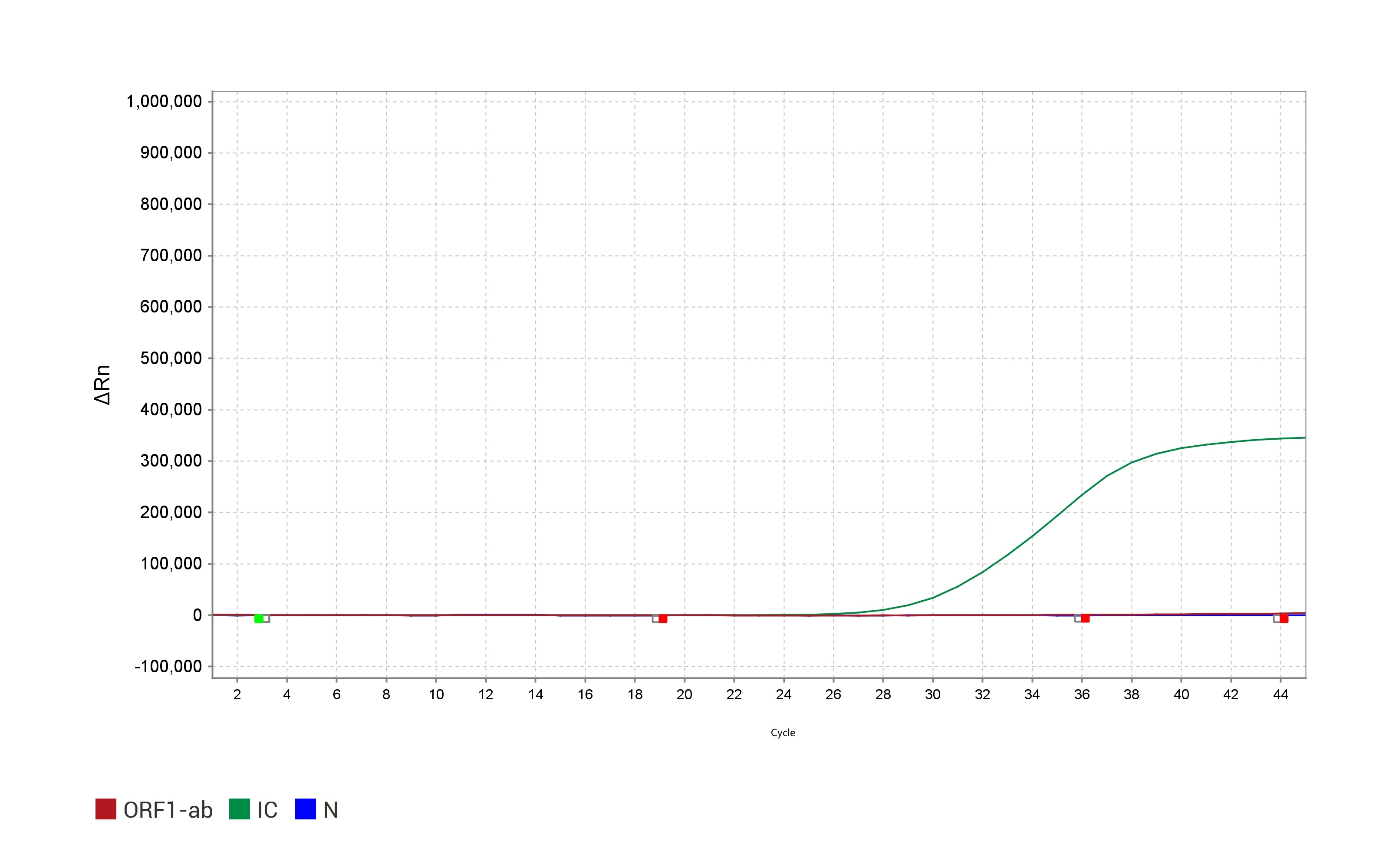

Supplement: S1 File — (ZIP) [file pone.0286121.s001.zip › DNA amplification graphs English/general ward Contaminated area Personal protective 37.3.jpg]

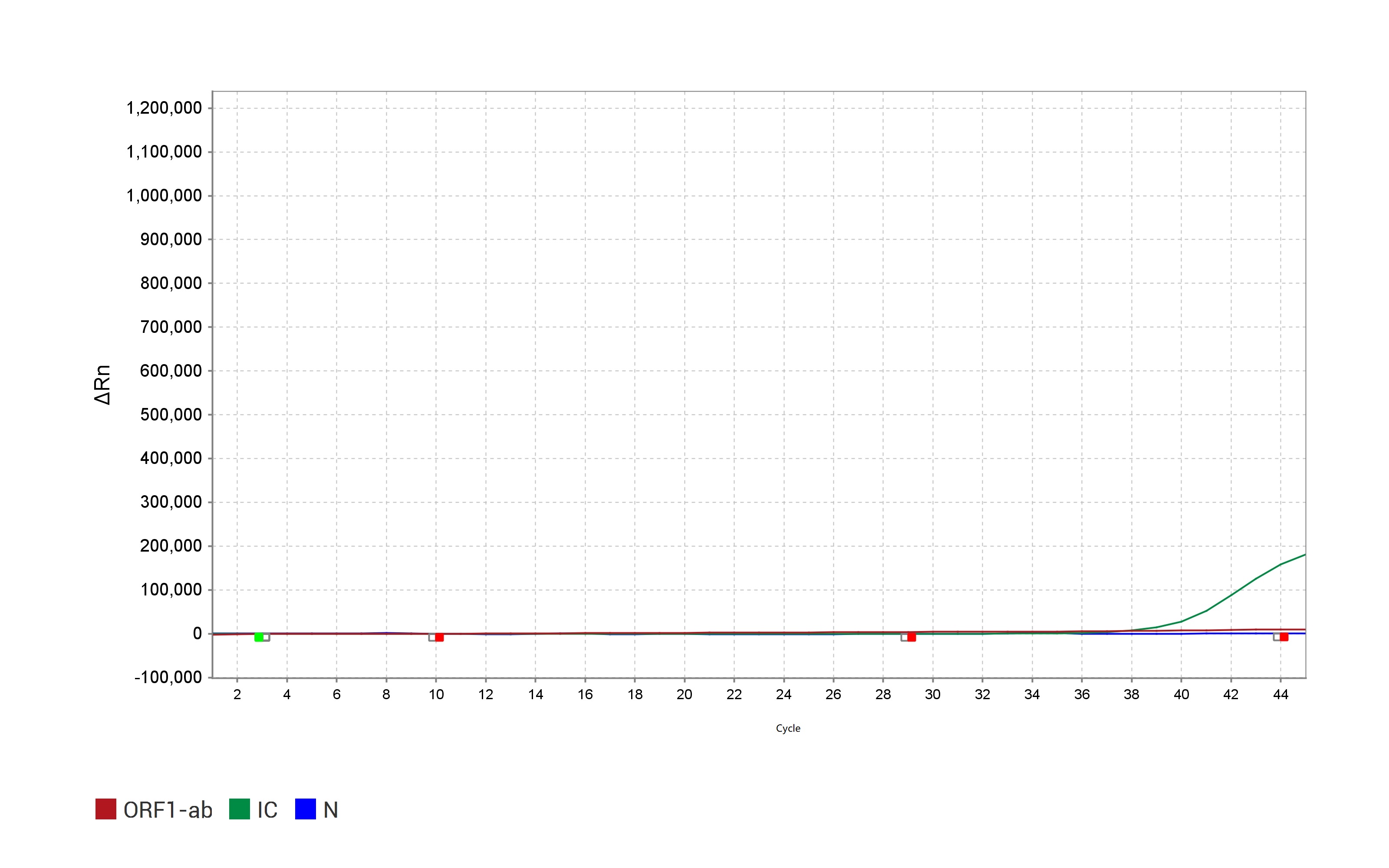

Supplement: S1 File — (ZIP) [file pone.0286121.s001.zip › DNA amplification graphs English/general ward Contaminated area Personal protective 39.0.jpg]

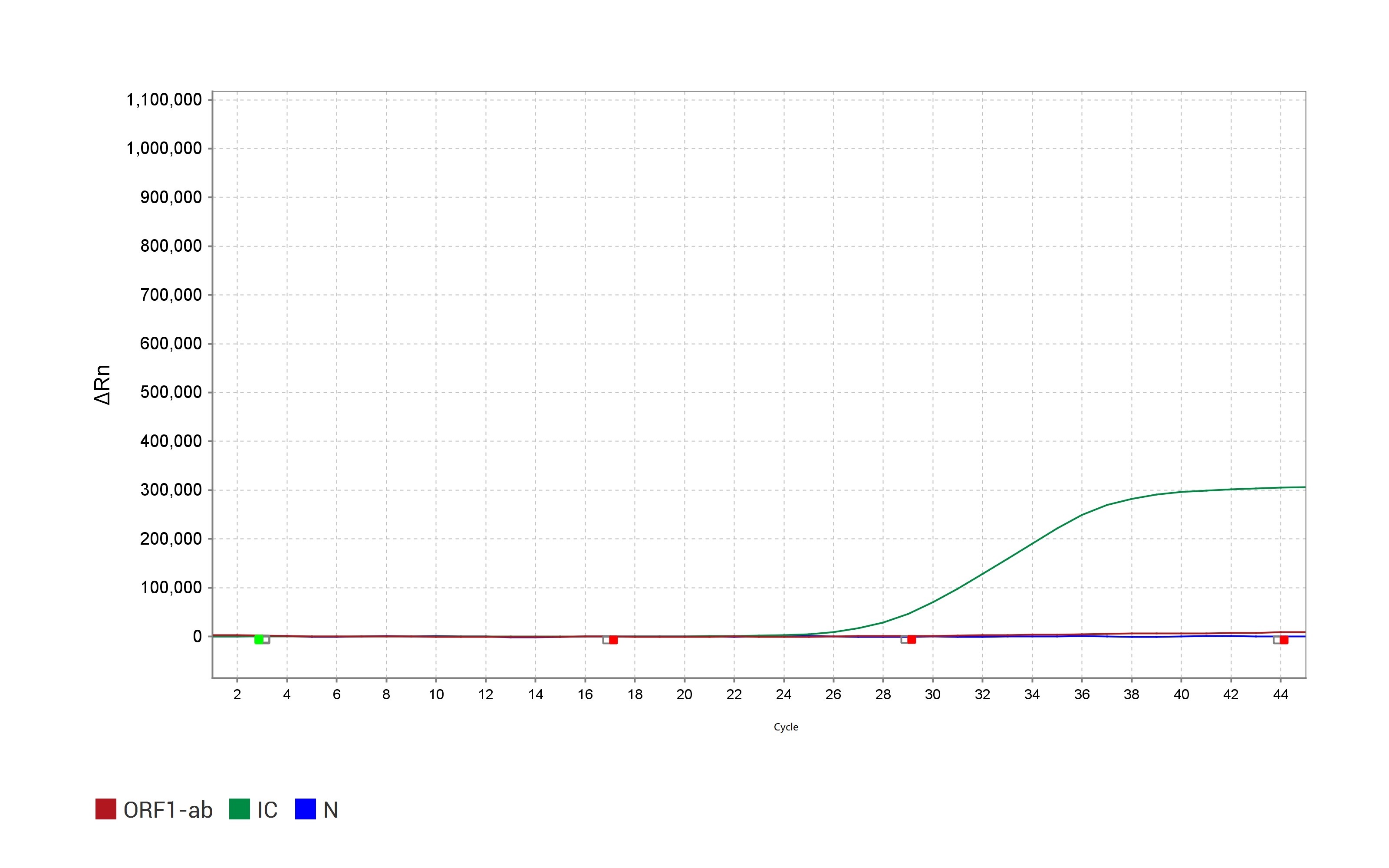

Supplement: S1 File — (ZIP) [file pone.0286121.s001.zip › DNA amplification graphs English/general ward Contaminated area Pillow 30.9.jpg]

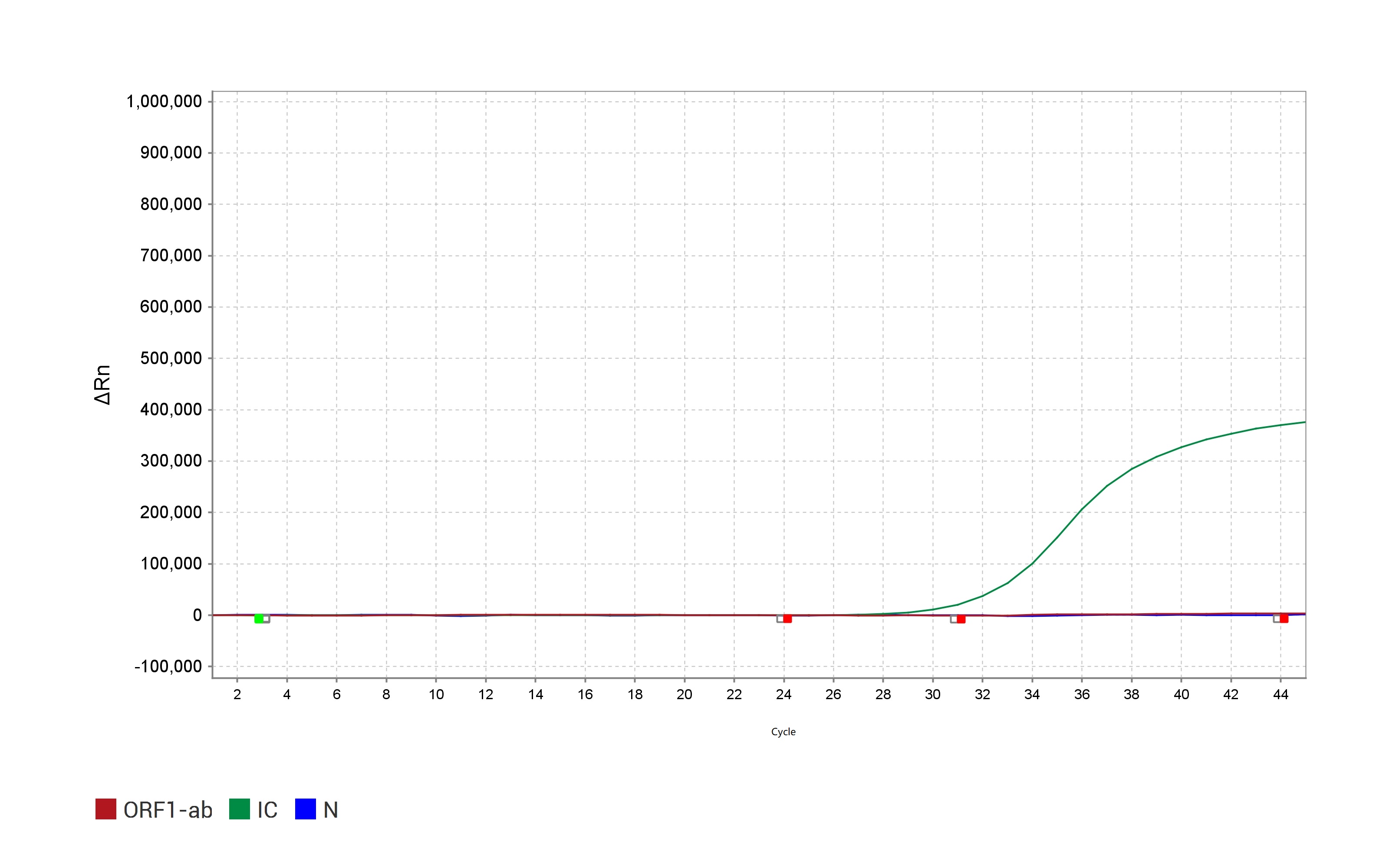

Supplement: S1 File — (ZIP) [file pone.0286121.s001.zip › DNA amplification graphs English/general ward Contaminated area Pillow 34.6.jpg]

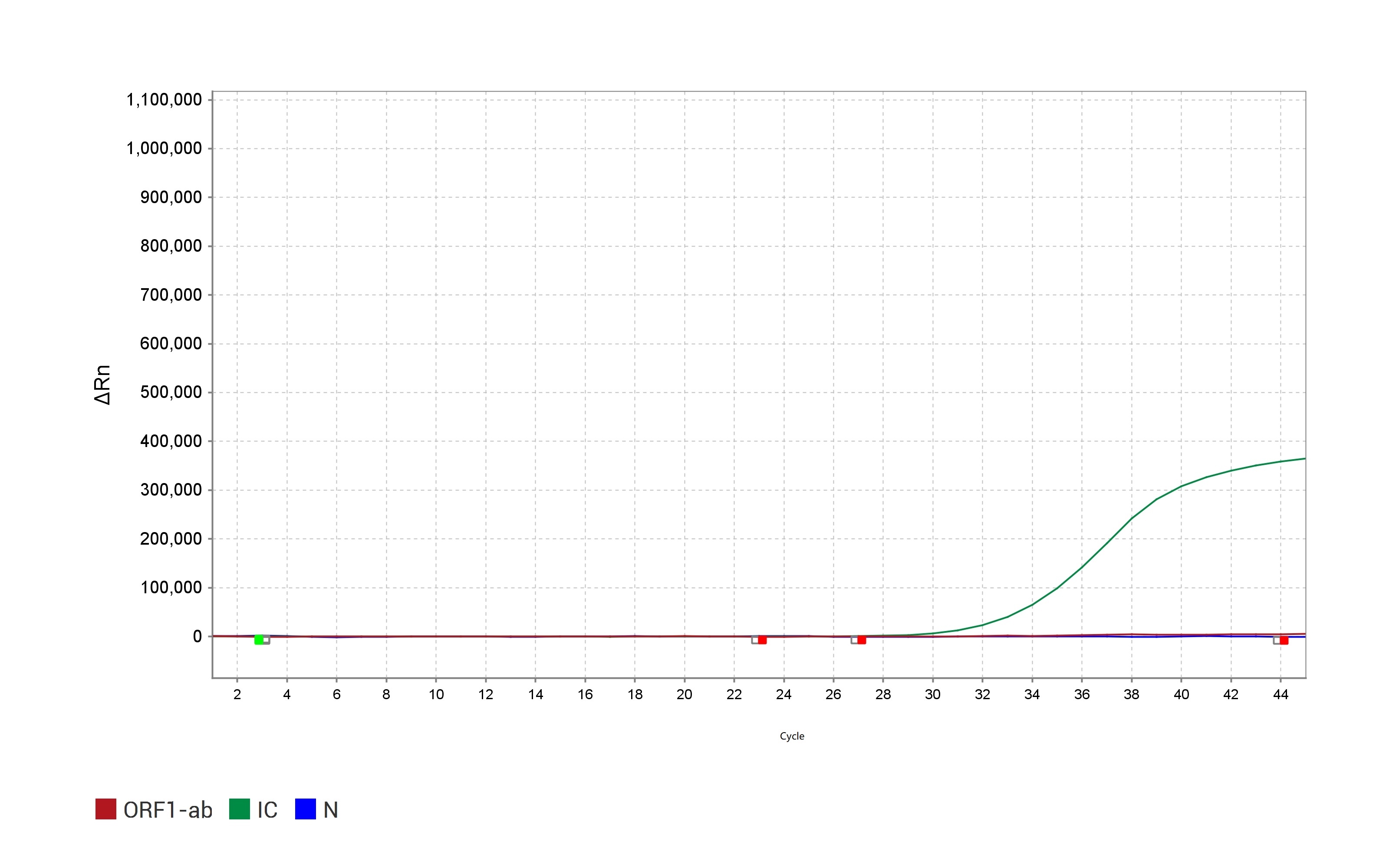

Supplement: S1 File — (ZIP) [file pone.0286121.s001.zip › DNA amplification graphs English/general ward Contaminated area Pillow 34.7.jpg]

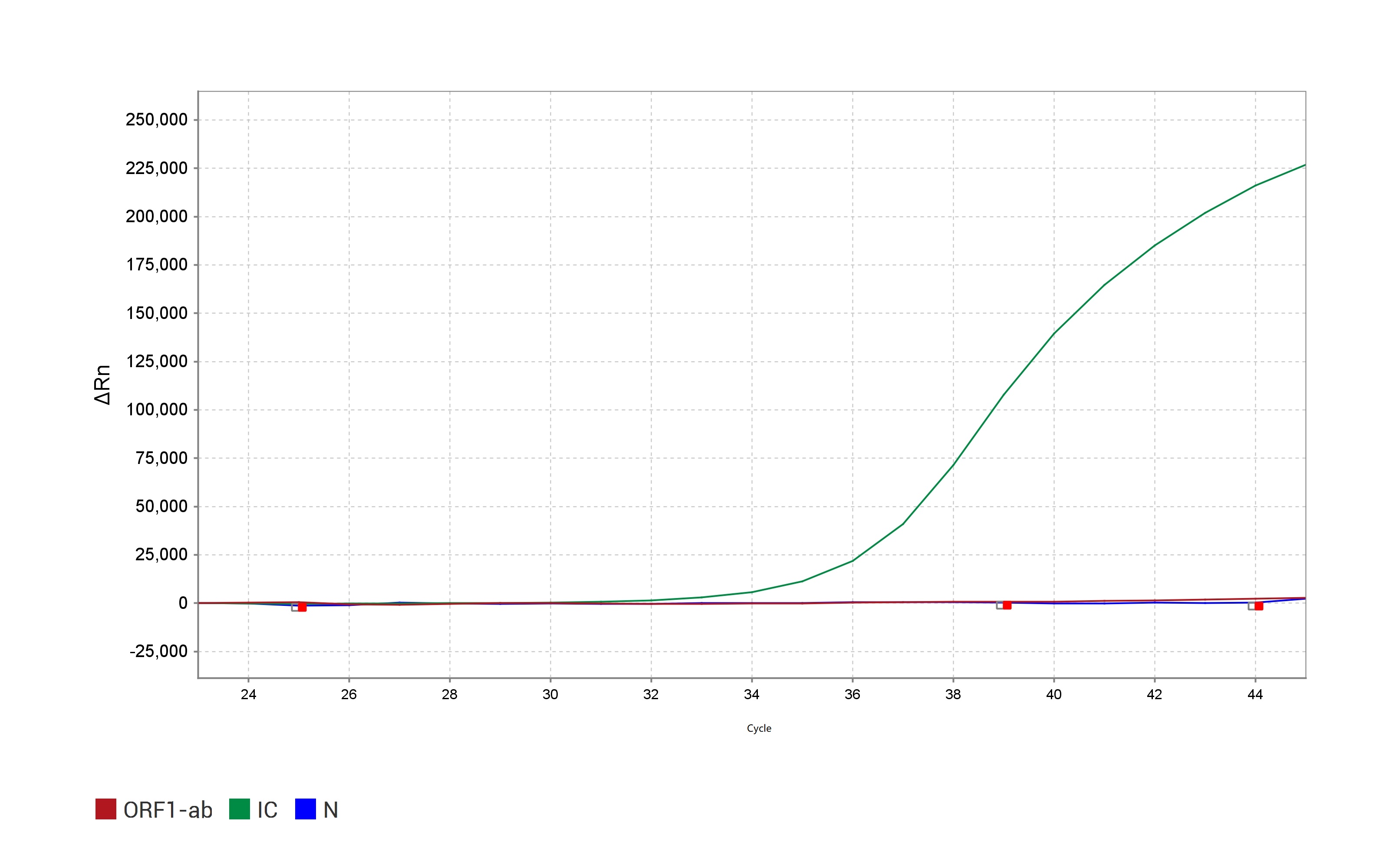

Supplement: S1 File — (ZIP) [file pone.0286121.s001.zip › DNA amplification graphs English/general ward Semi-contaminated area Door handle 39.0.jpg]

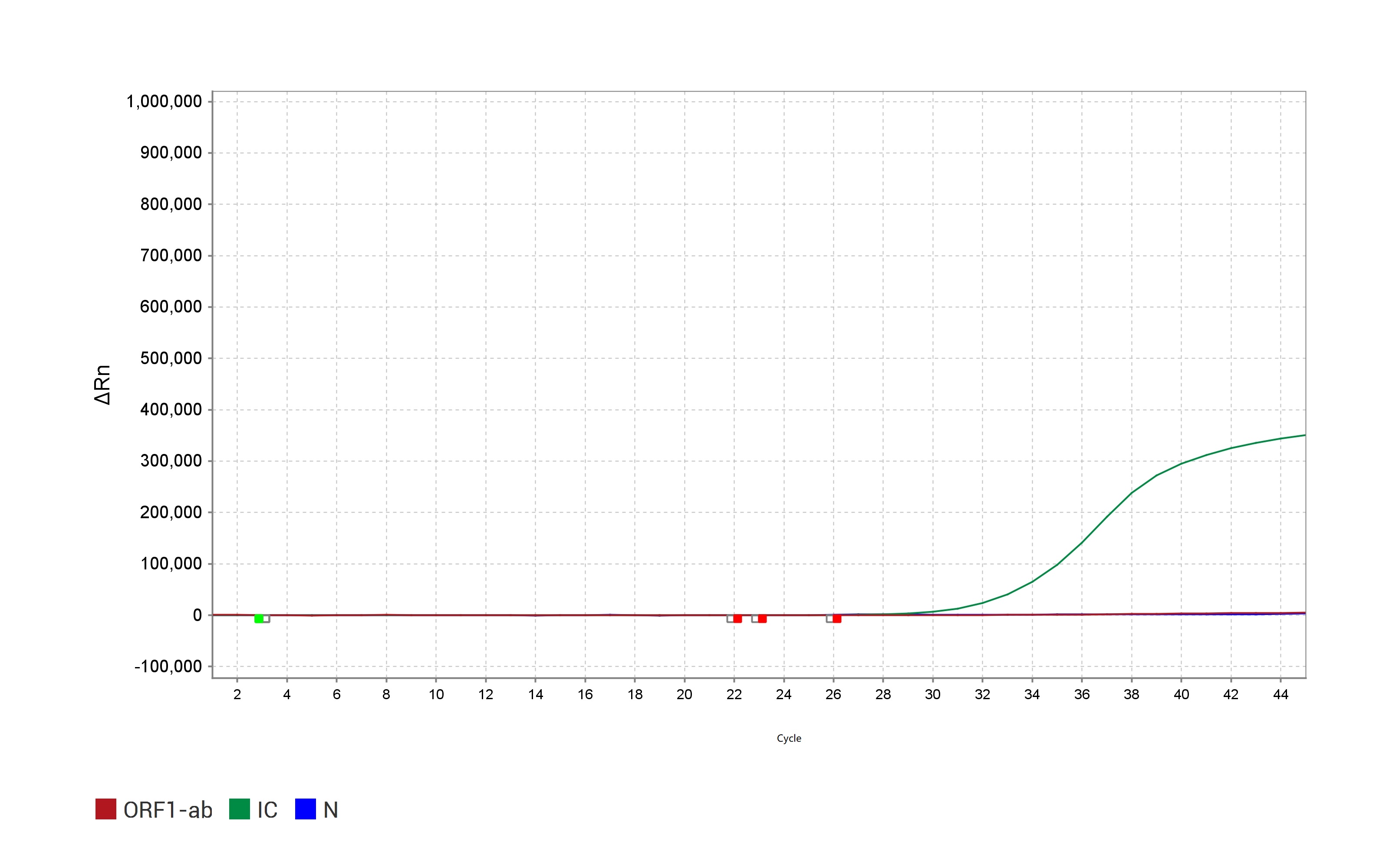

Supplement: S1 File — (ZIP) [file pone.0286121.s001.zip › DNA amplification graphs English/intensive care unit Contaminated area Computer keyboard of nurse station 35.0 35.6.jpg]

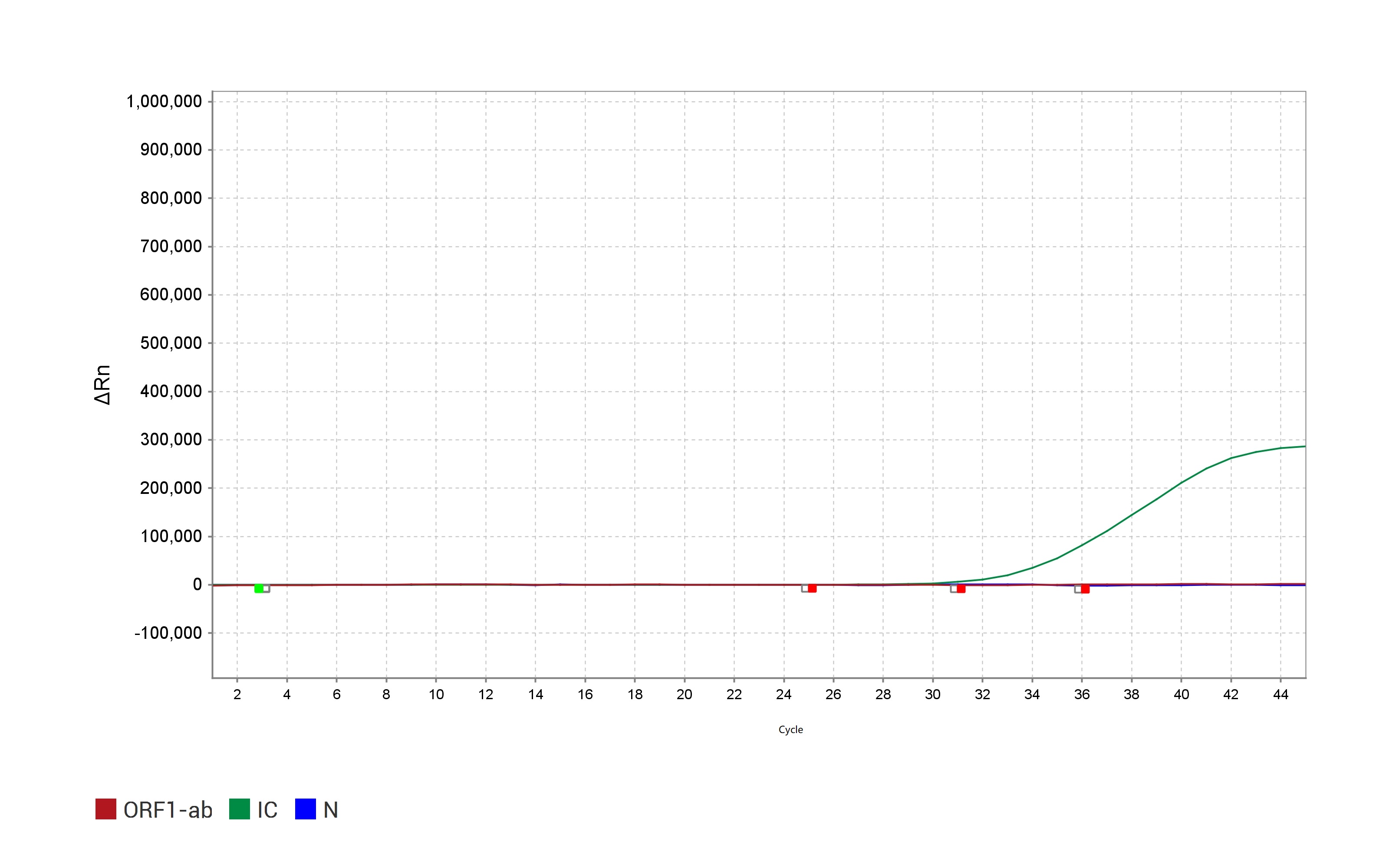

Supplement: S1 File — (ZIP) [file pone.0286121.s001.zip › DNA amplification graphs English/intensive care unit Contaminated area Computer keyboard of nurse station 37.8 37.5.jpg]

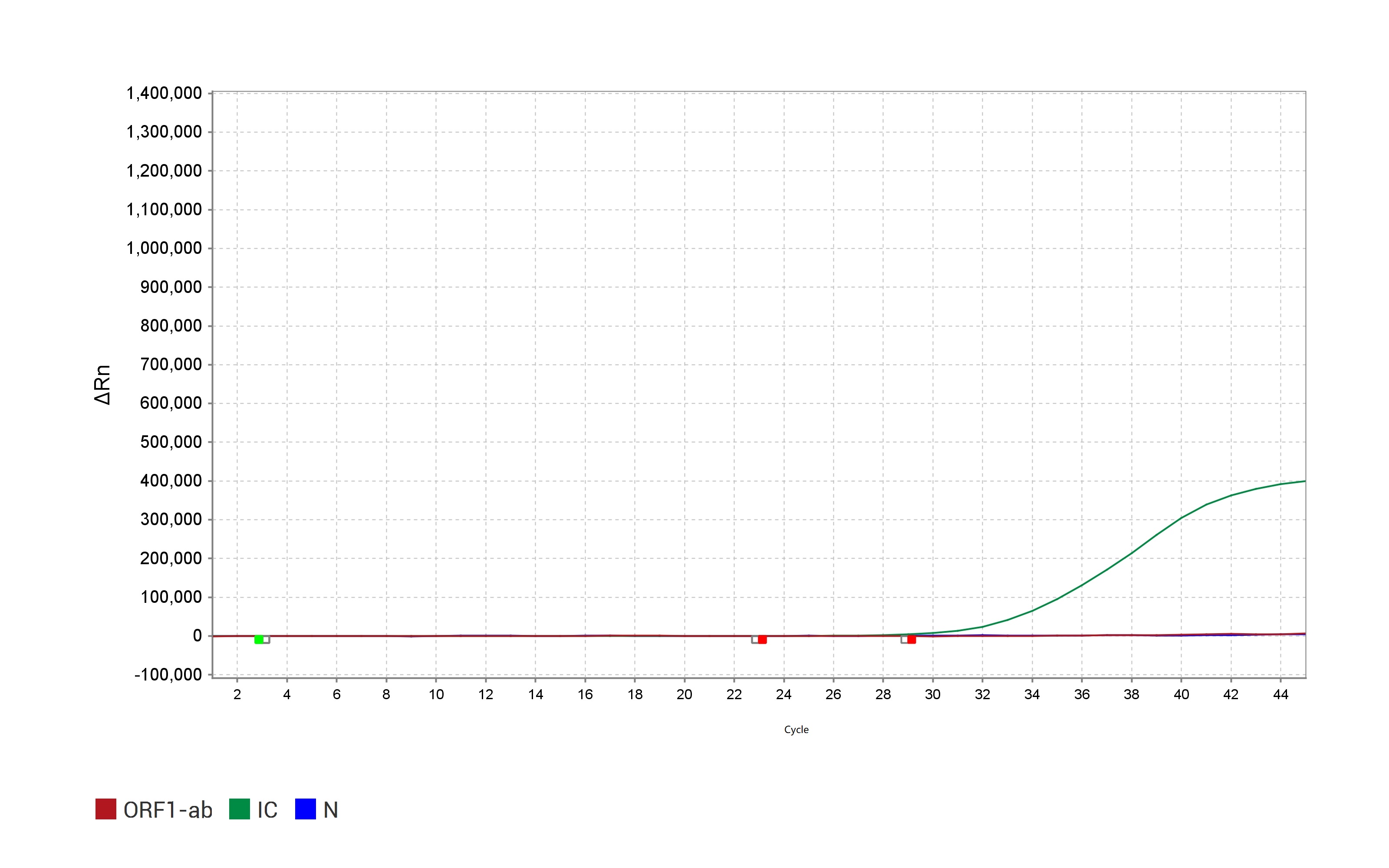

Supplement: S1 File — (ZIP) [file pone.0286121.s001.zip › DNA amplification graphs English/intensive care unit Contaminated area Medical equipment 35.4 34.6.jpg]

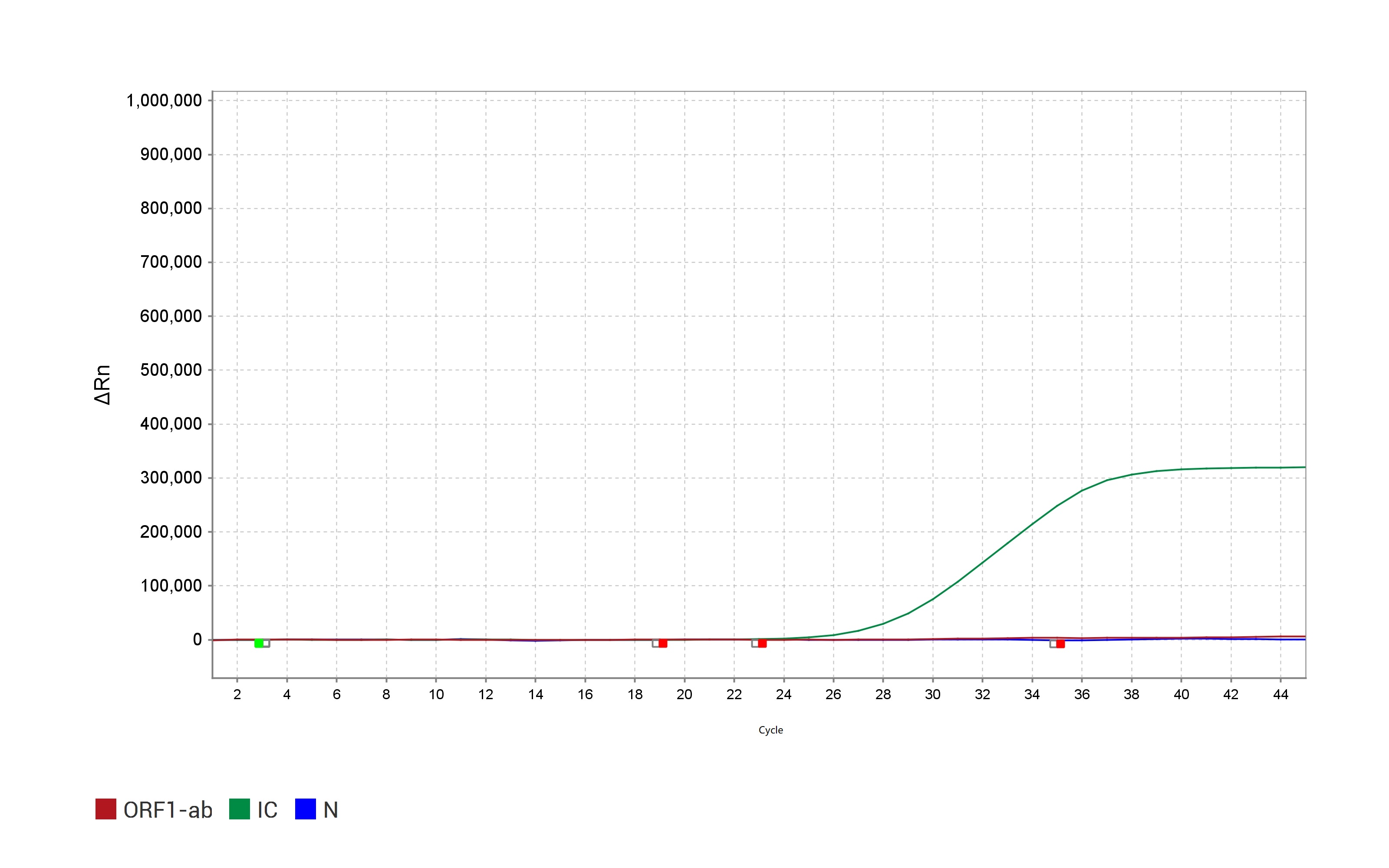

Supplement: S1 File — (ZIP) [file pone.0286121.s001.zip › DNA amplification graphs English/intensive care unit Semi-contaminated area Door handle 39.5 37.7.jpg]

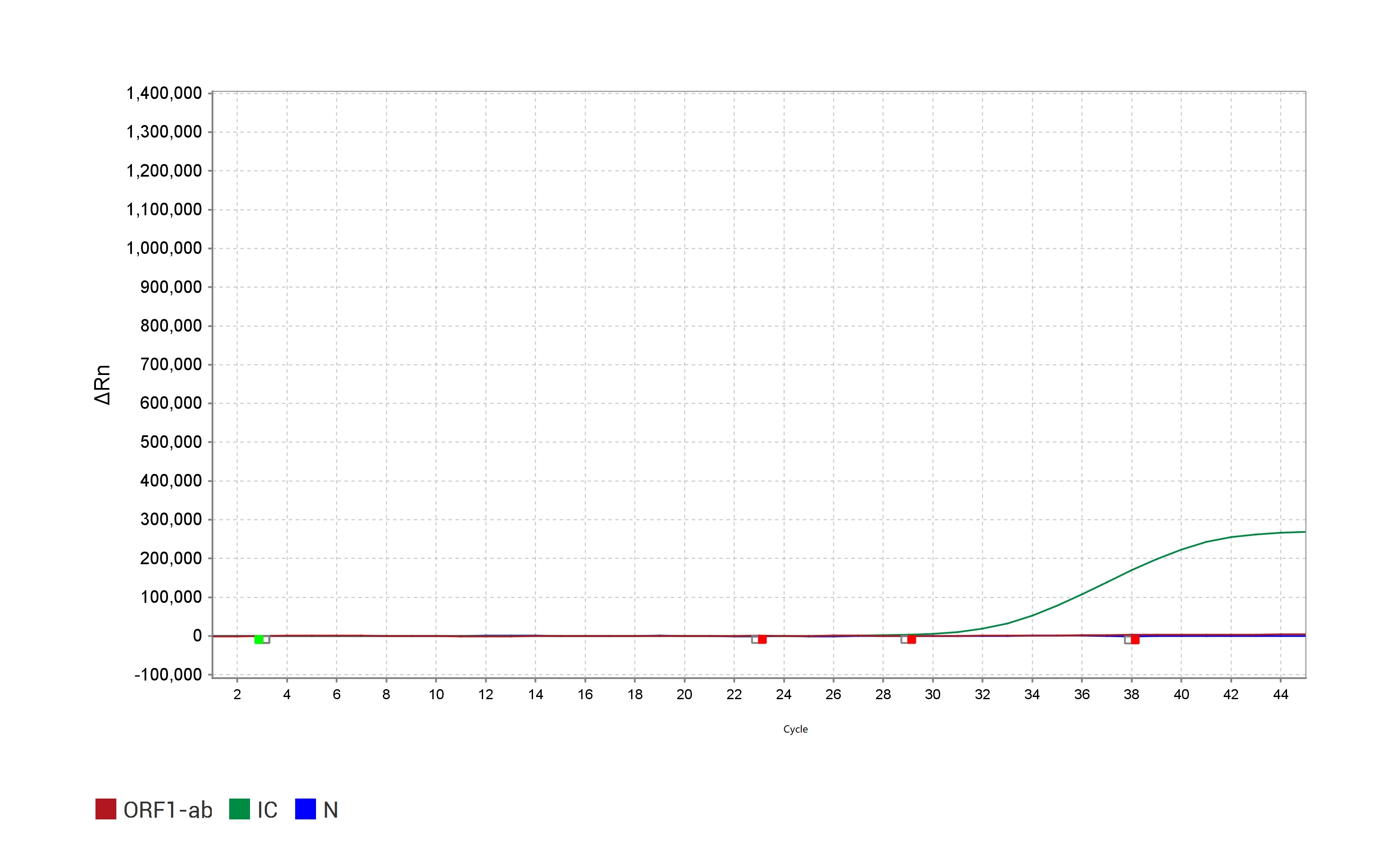

Supplement: S1 File — (ZIP) [file pone.0286121.s001.zip › DNA amplification graphs English/intensive care unit Clean area Door handle 37.0 39.8.jpg]

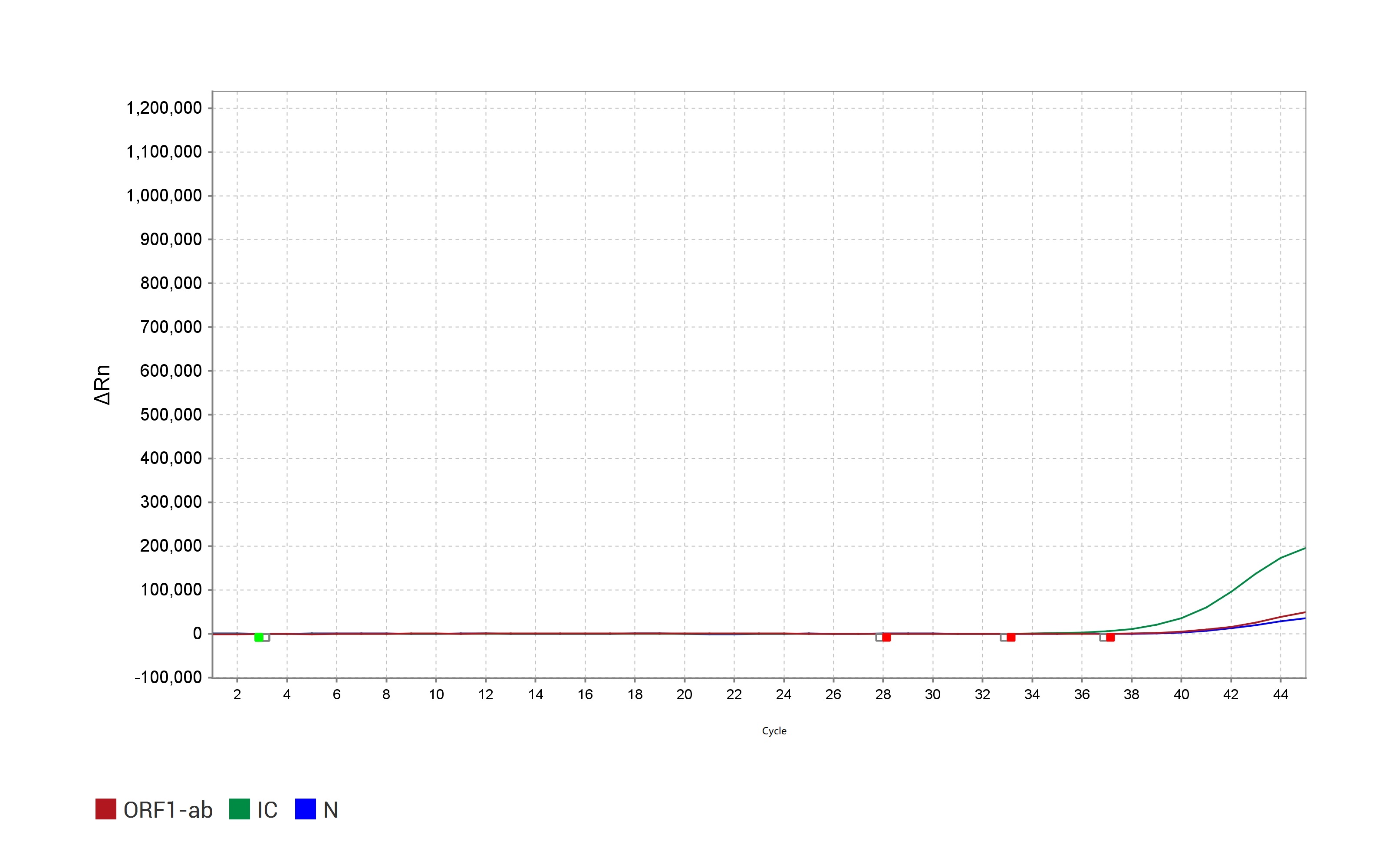

Supplement: S1 File — (ZIP) [file pone.0286121.s001.zip › DNA amplification graphs English/intensive care unit Contaminated area Computer keyboard of nurse station 37.8 37.8.jpg]

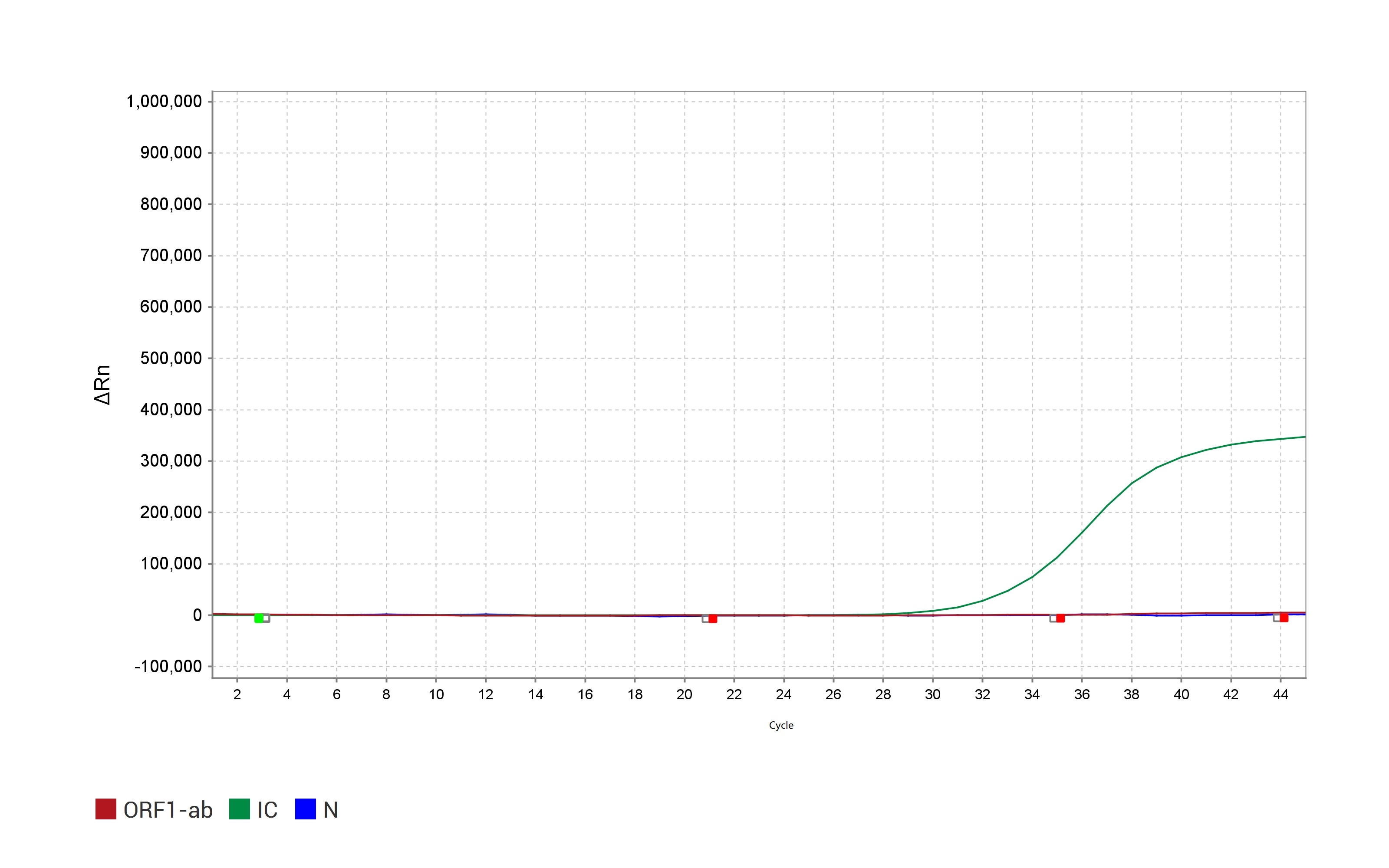

Supplement: S1 File — (ZIP) [file pone.0286121.s001.zip › DNA amplification graphs English/intensive care unit Contaminated area Door handle 33.709.jpg]

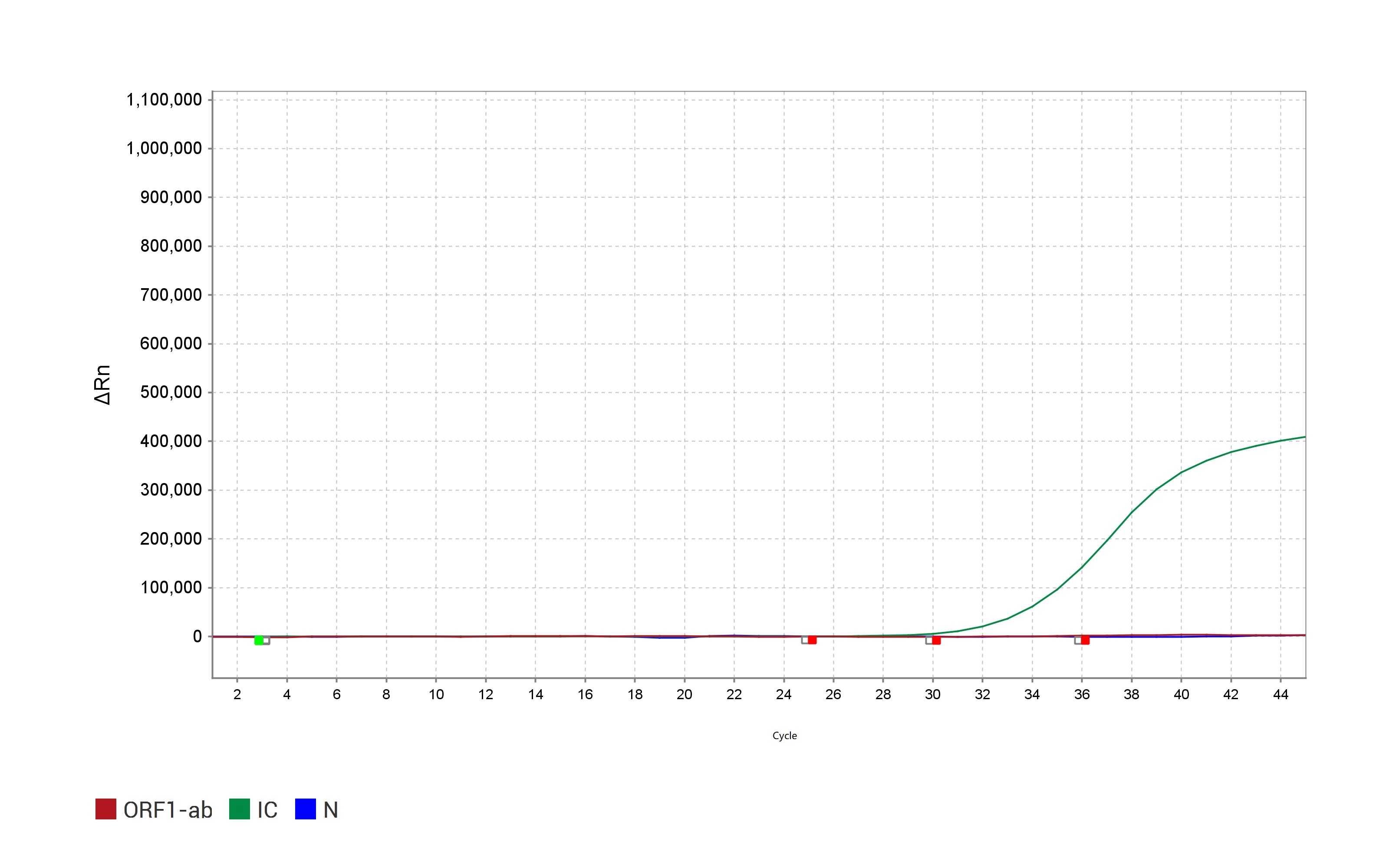

Supplement: S1 File — (ZIP) [file pone.0286121.s001.zip › DNA amplification graphs English/intensive care unit Contaminated area Door handle 35.4 36.4.jpg]

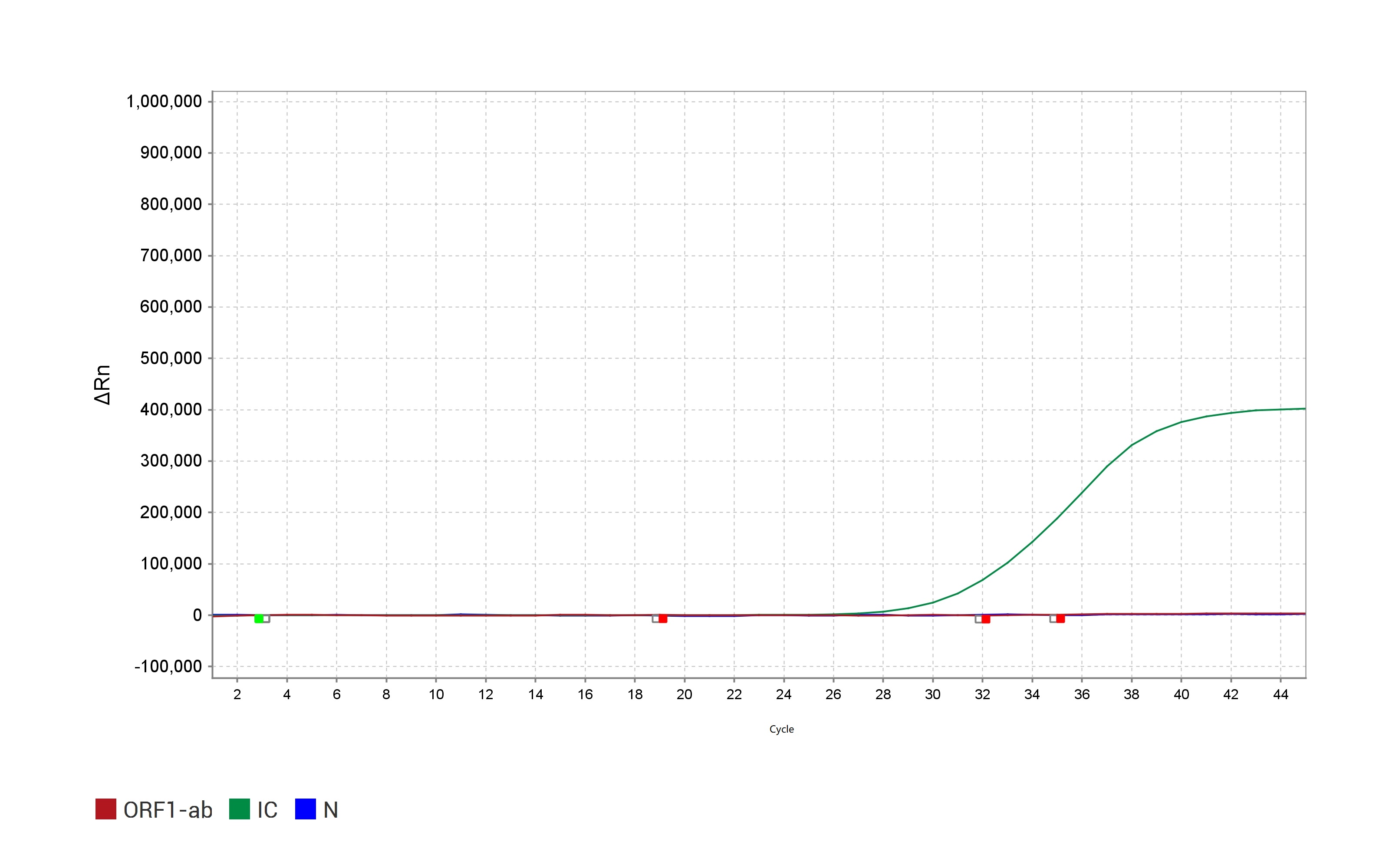

Supplement: S1 File — (ZIP) [file pone.0286121.s001.zip › DNA amplification graphs English/intensive care unit Contaminated area Door handle 36.3 35.2.jpg]

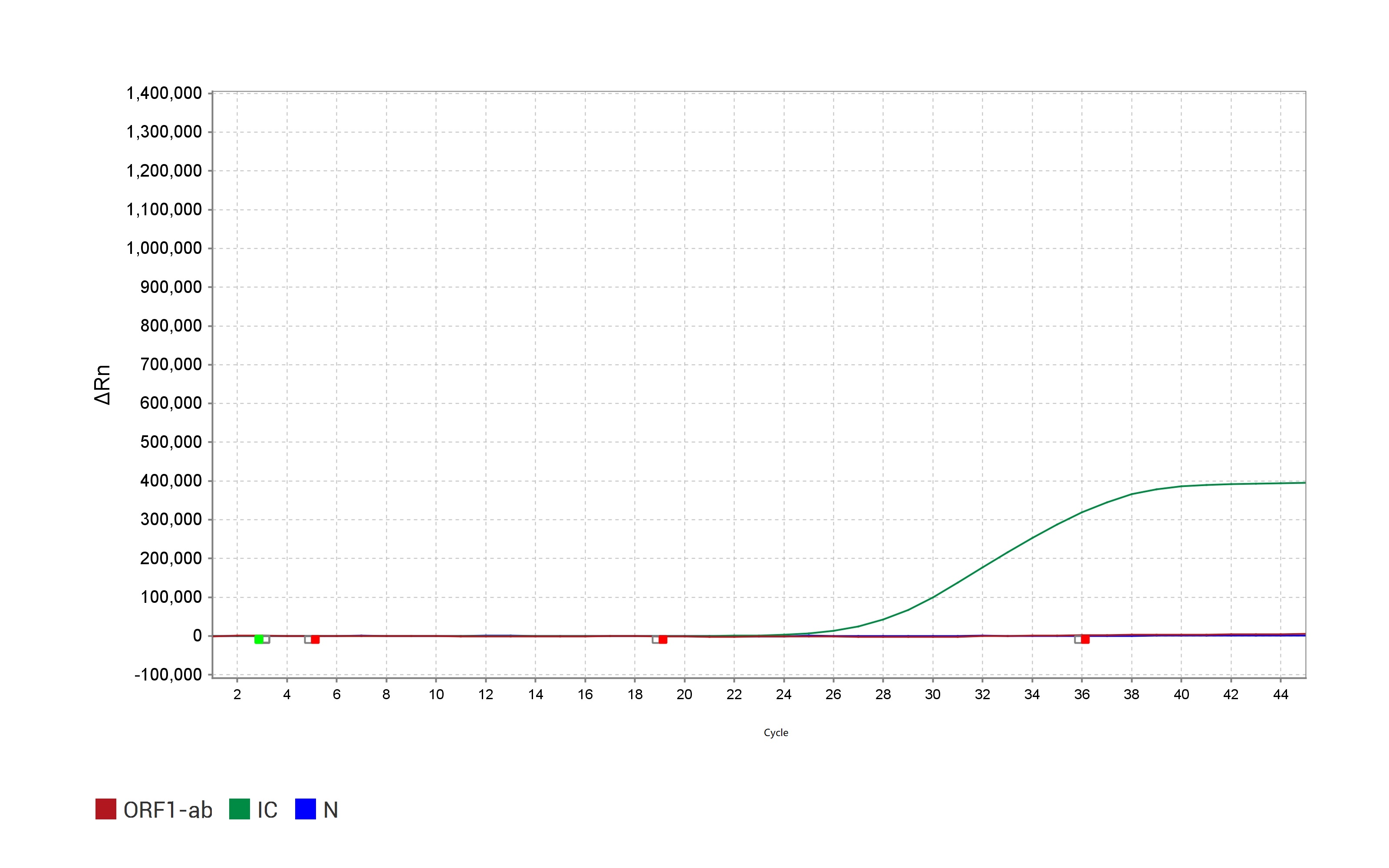

Supplement: S1 File — (ZIP) [file pone.0286121.s001.zip › DNA amplification graphs English/intensive care unit Contaminated area Door handle35.4 36.4.jpg]

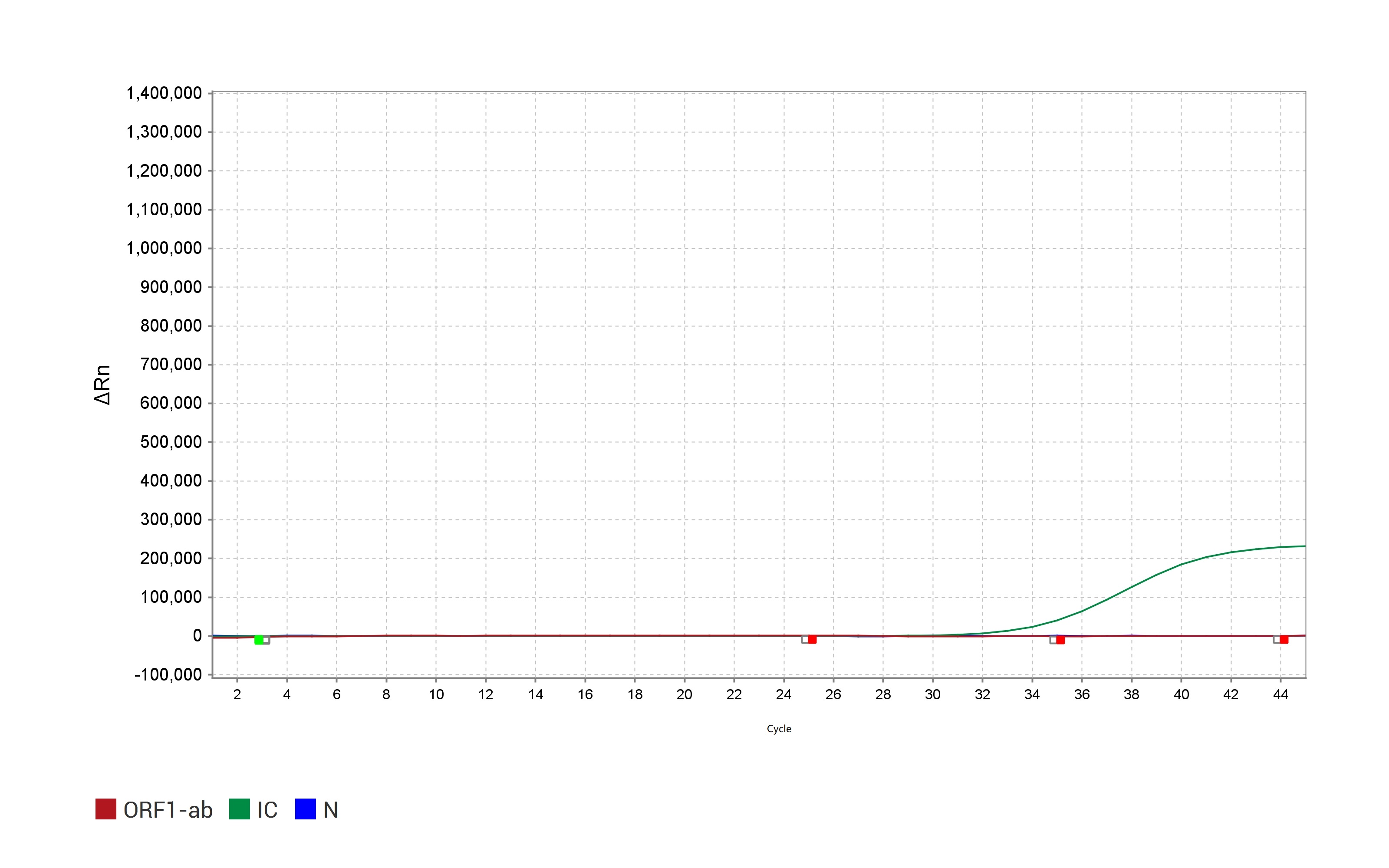

Supplement: S1 File — (ZIP) [file pone.0286121.s001.zip › DNA amplification graphs English/intensive care unit Contaminated area Medical equipment 39.7 40.1.jpg]

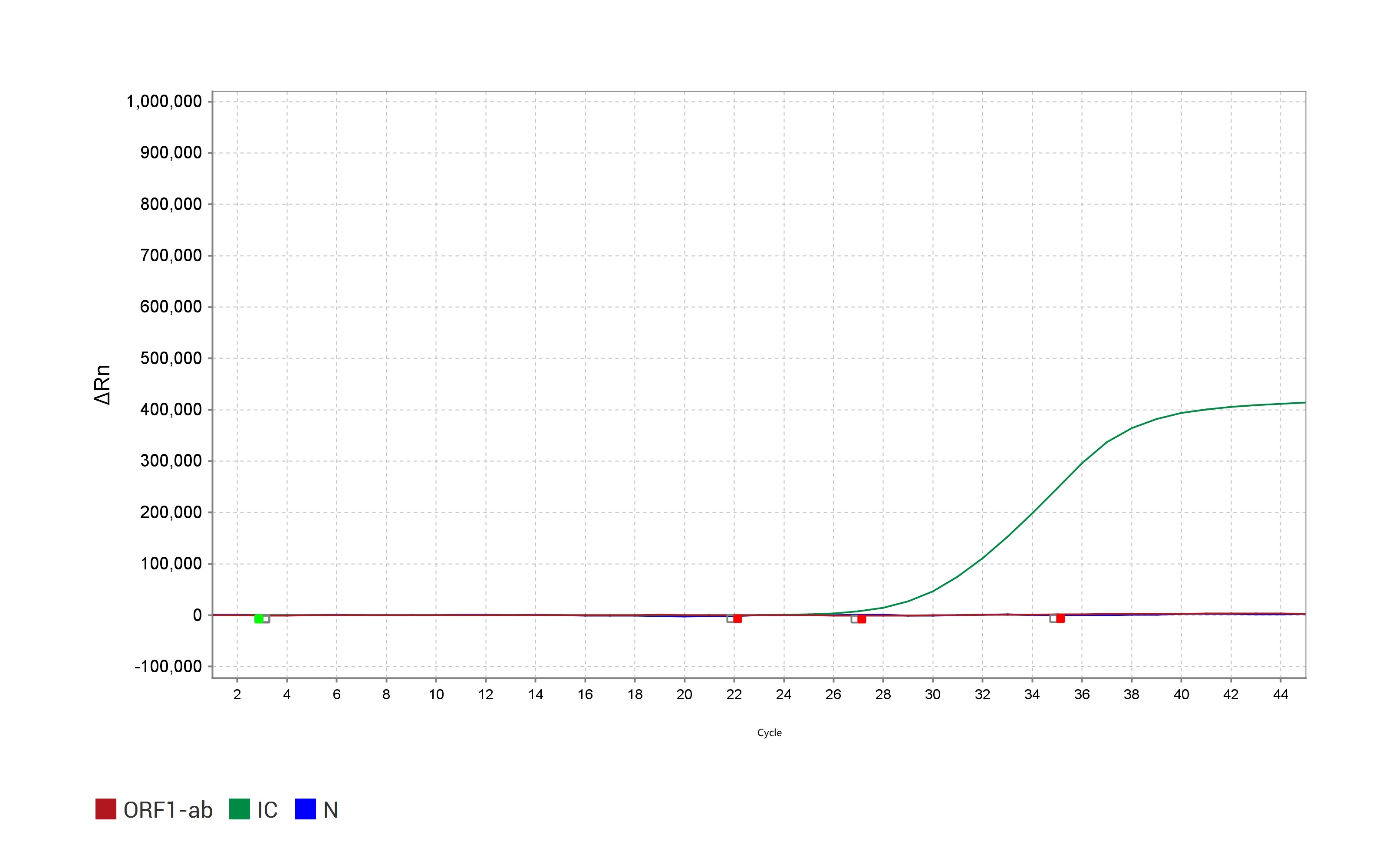

Supplement: S1 File — (ZIP) [file pone.0286121.s001.zip › DNA amplification graphs English/intensive care unit Contaminated area Medical equipment 35.4 34.6.jpg]

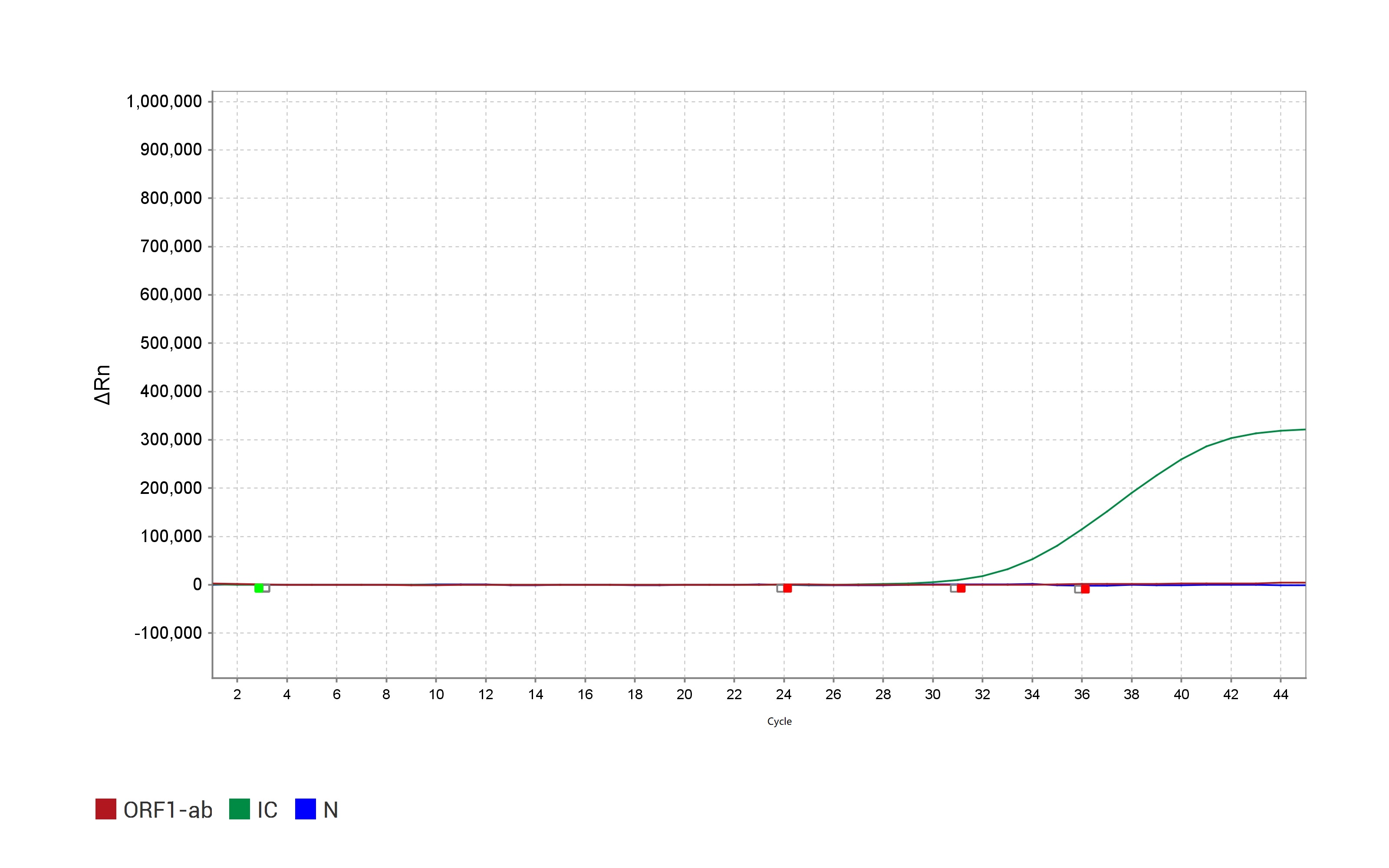

Supplement: S1 File — (ZIP) [file pone.0286121.s001.zip › DNA amplification graphs English/intensive care unit Contaminated area Medical equipment 39.7 40.1.jpg]

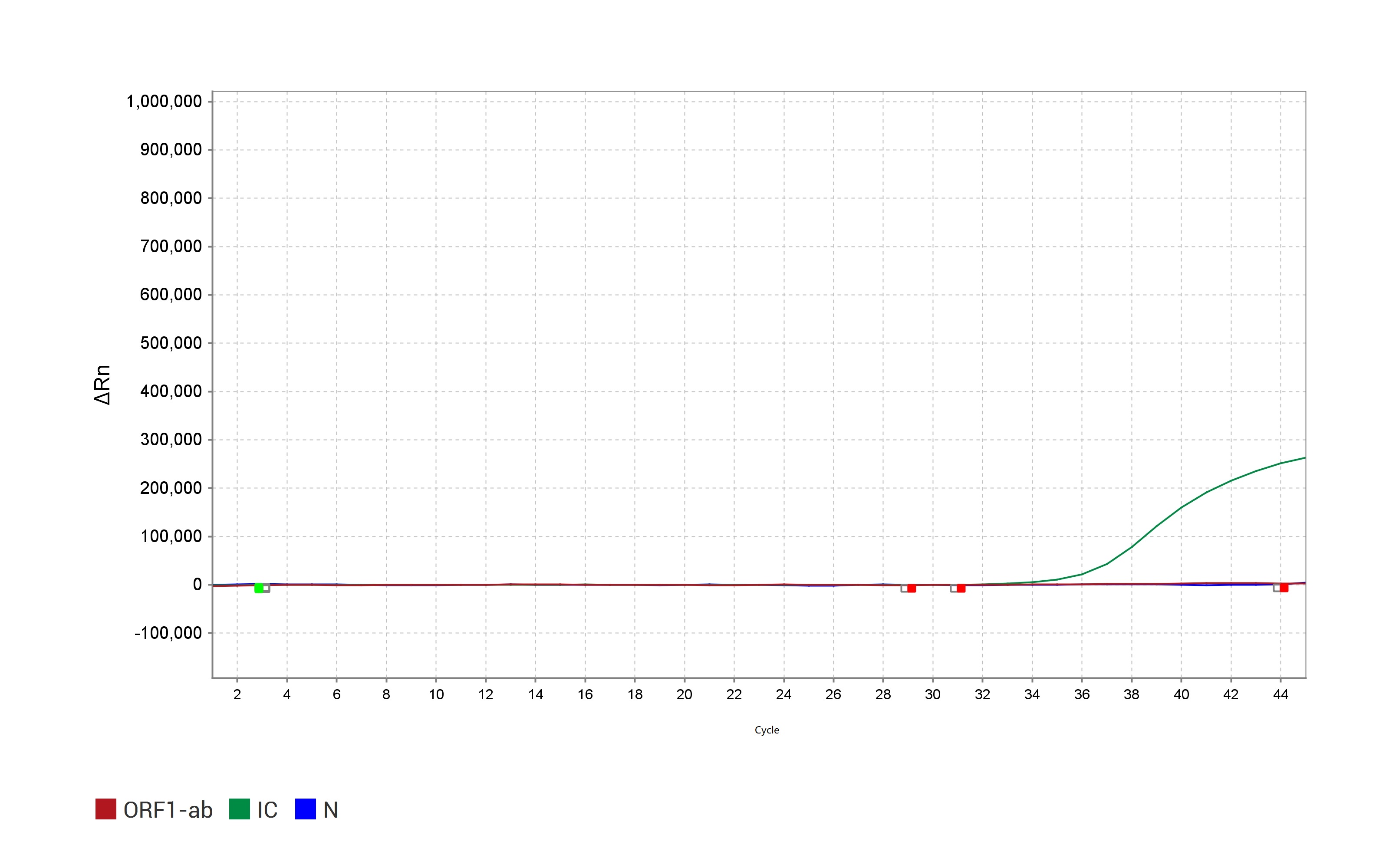

Supplement: S1 File — (ZIP) [file pone.0286121.s001.zip › DNA amplification graphs English/intensive care unit Contaminated area Nurse station stable 35.0 35.6.jpg]

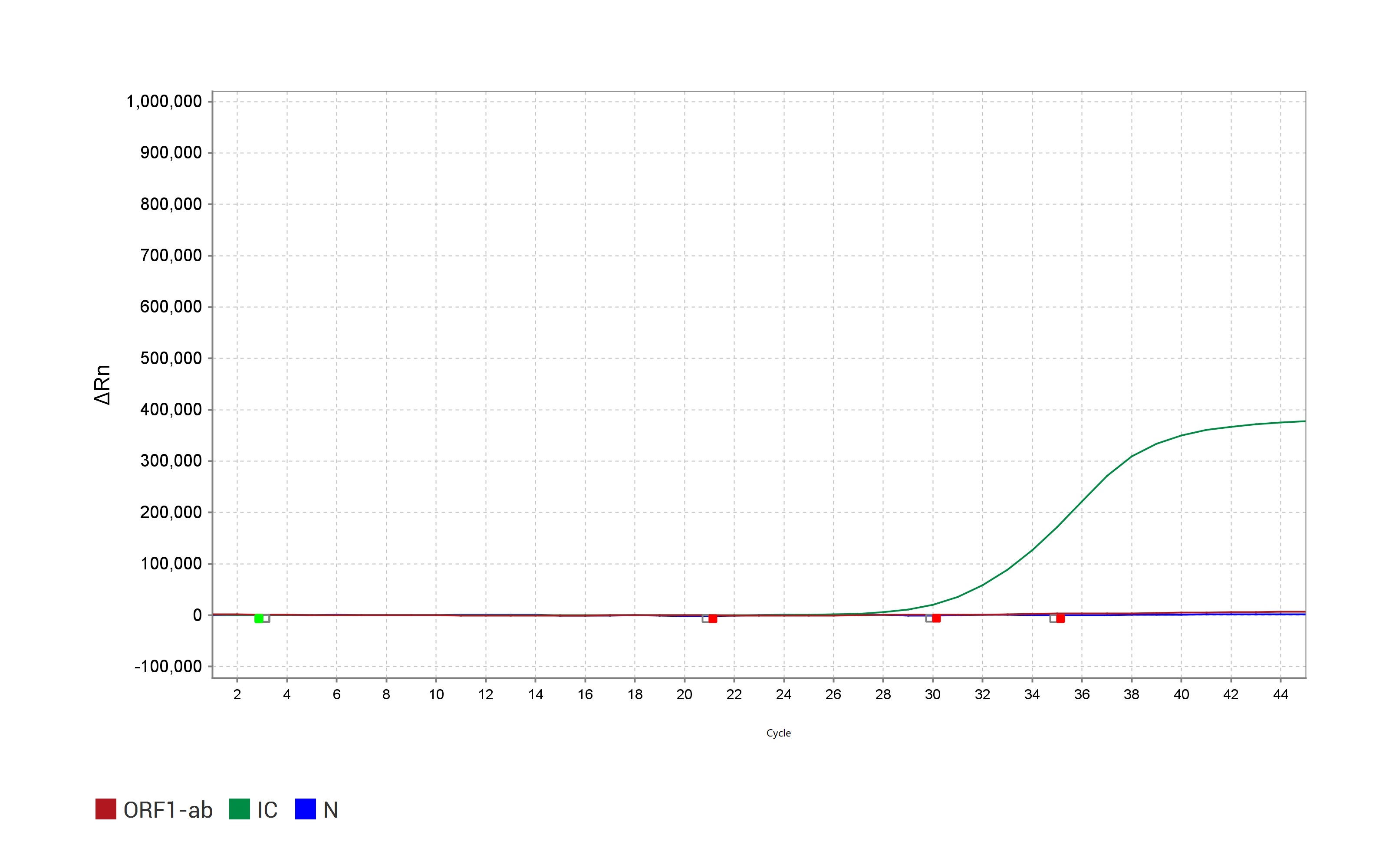

Supplement: S1 File — (ZIP) [file pone.0286121.s001.zip › DNA amplification graphs English/intensive care unit Contaminated area Nurse station stable 34.8 35.7 .jpg]

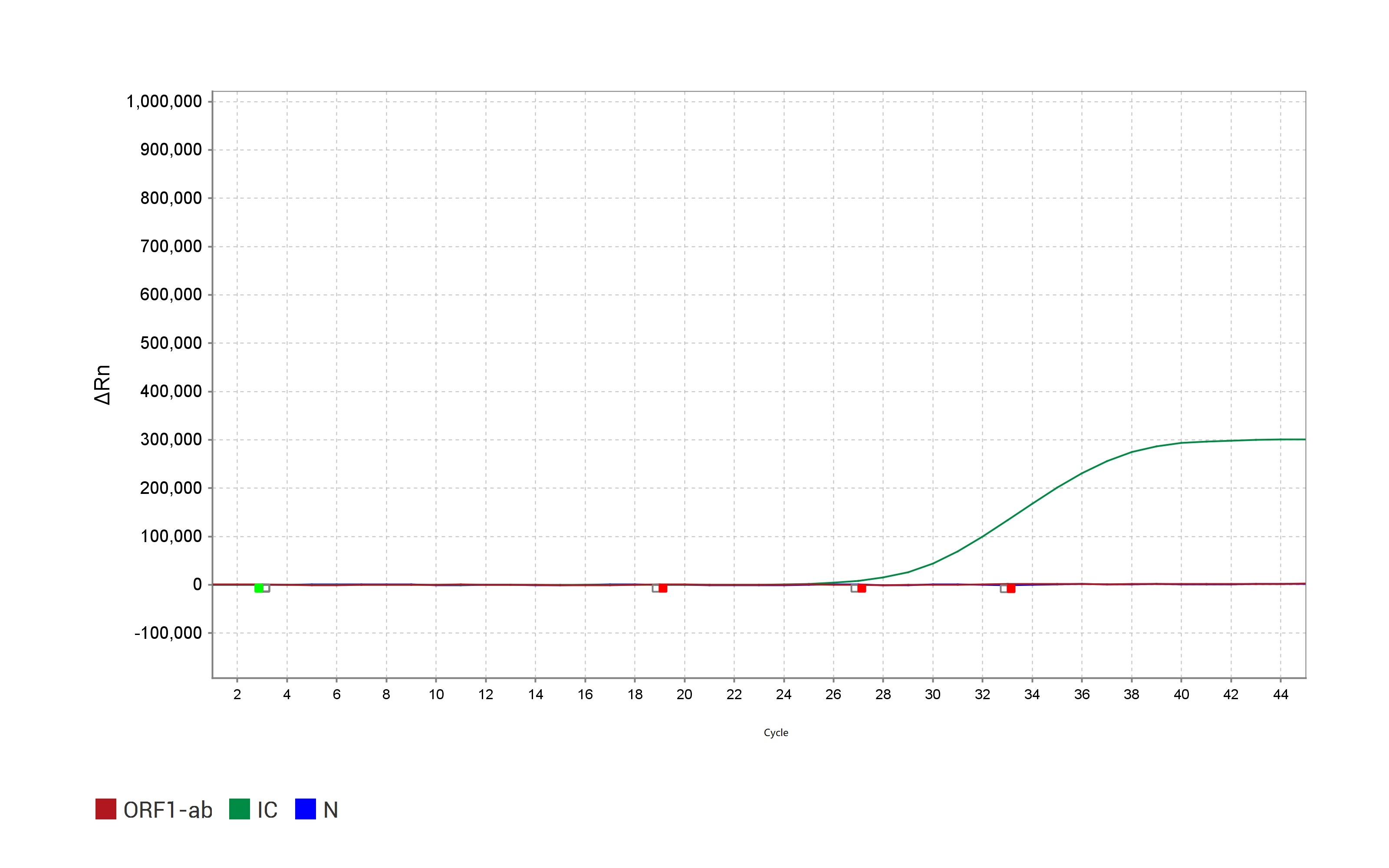

Supplement: S1 File — (ZIP) [file pone.0286121.s001.zip › DNA amplification graphs English/intensive care unit Contaminated area Nurse station stable 34.8 35.7.jpg]

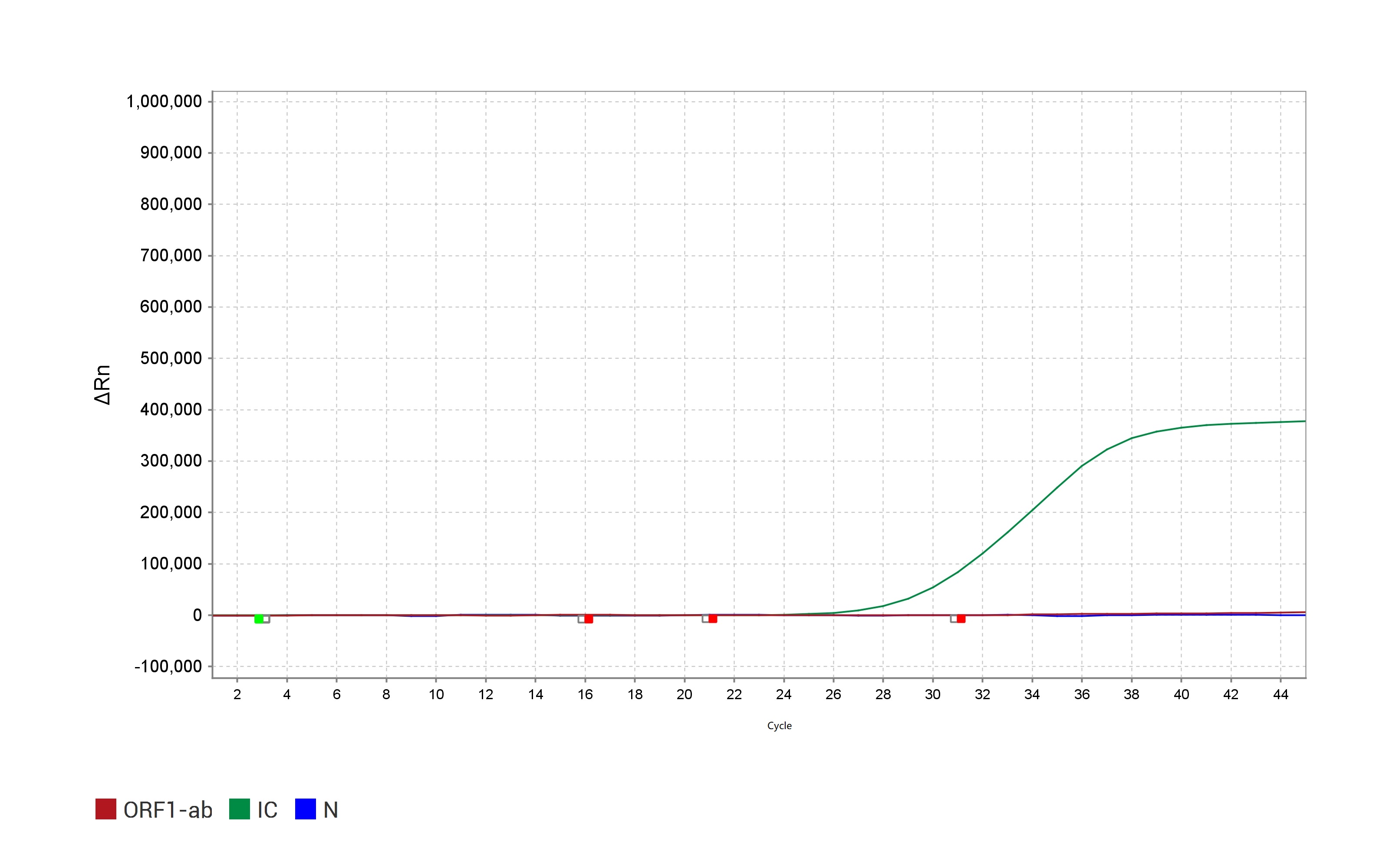

Supplement: S1 File — (ZIP) [file pone.0286121.s001.zip › DNA amplification graphs English/intensive care unit Contaminated area Nurse station stable 35.0 35.6.jpg]

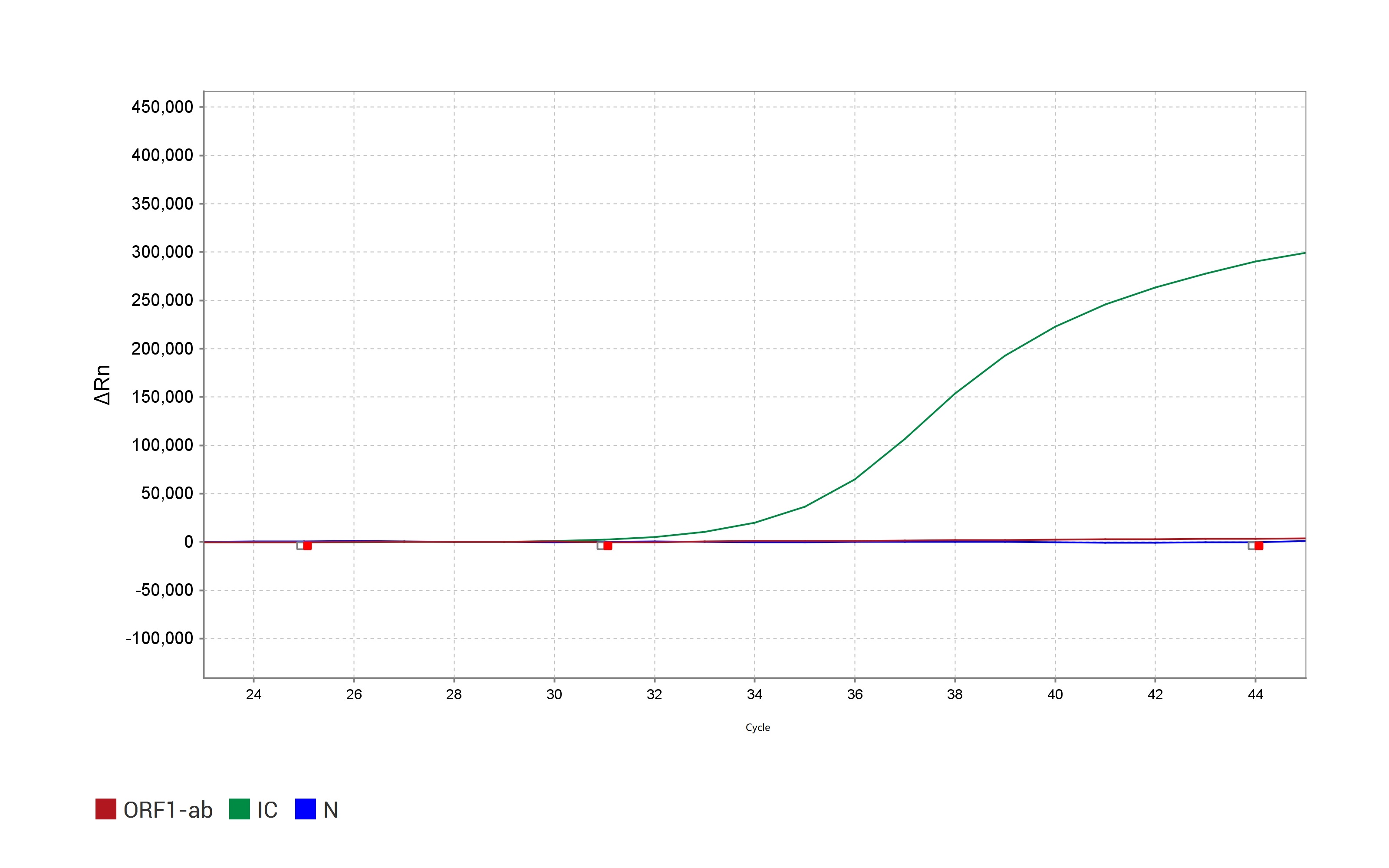

Supplement: S1 File — (ZIP) [file pone.0286121.s001.zip › DNA amplification graphs English/intensive care unit Semi-contaminated area Dispensing room table 34.3 35.2.jpg]

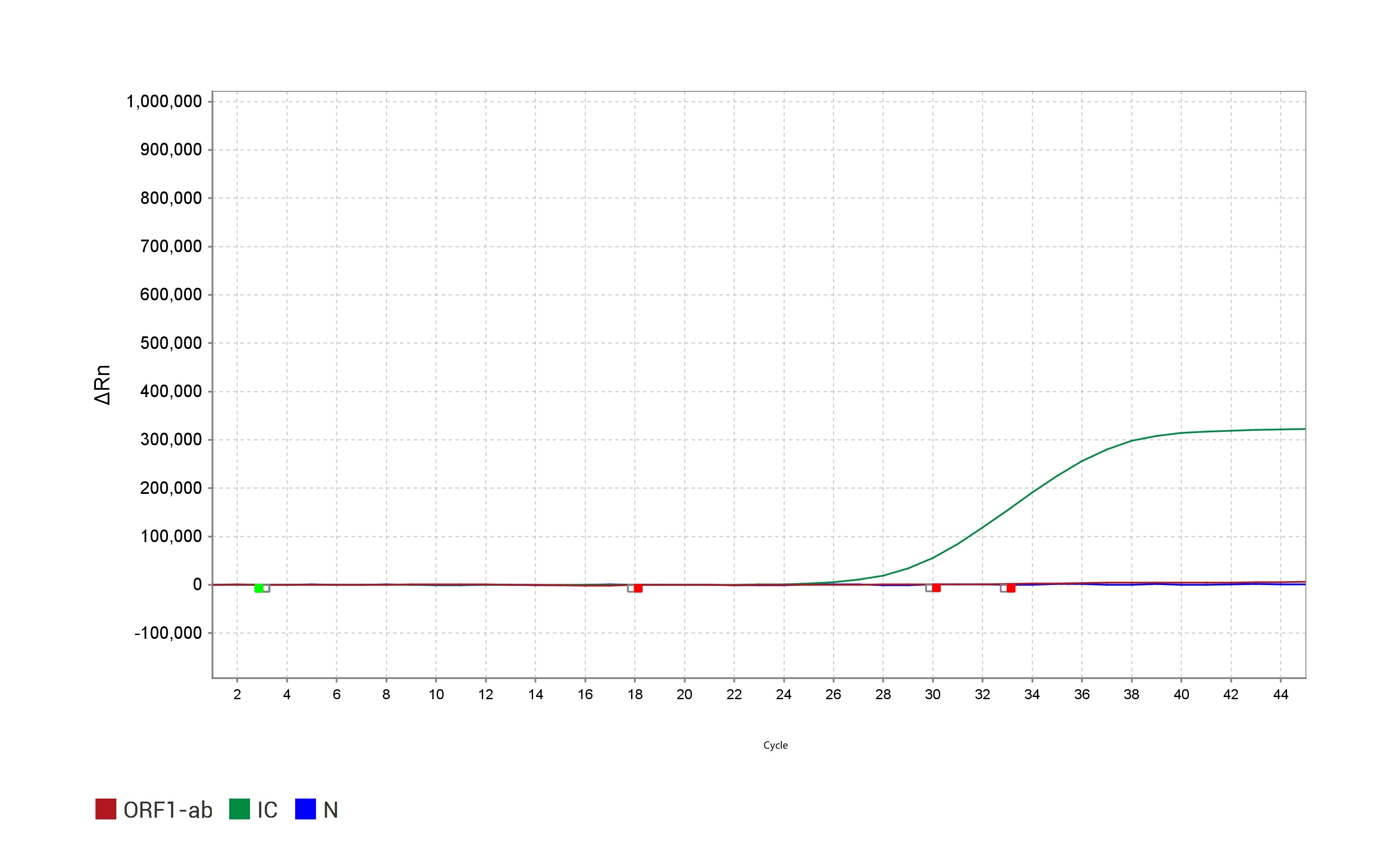

Supplement: S1 File — (ZIP) [file pone.0286121.s001.zip › DNA amplification graphs English/intensive care unit Semi-contaminated area Dispensing room table 34.3 35.2.jpg]

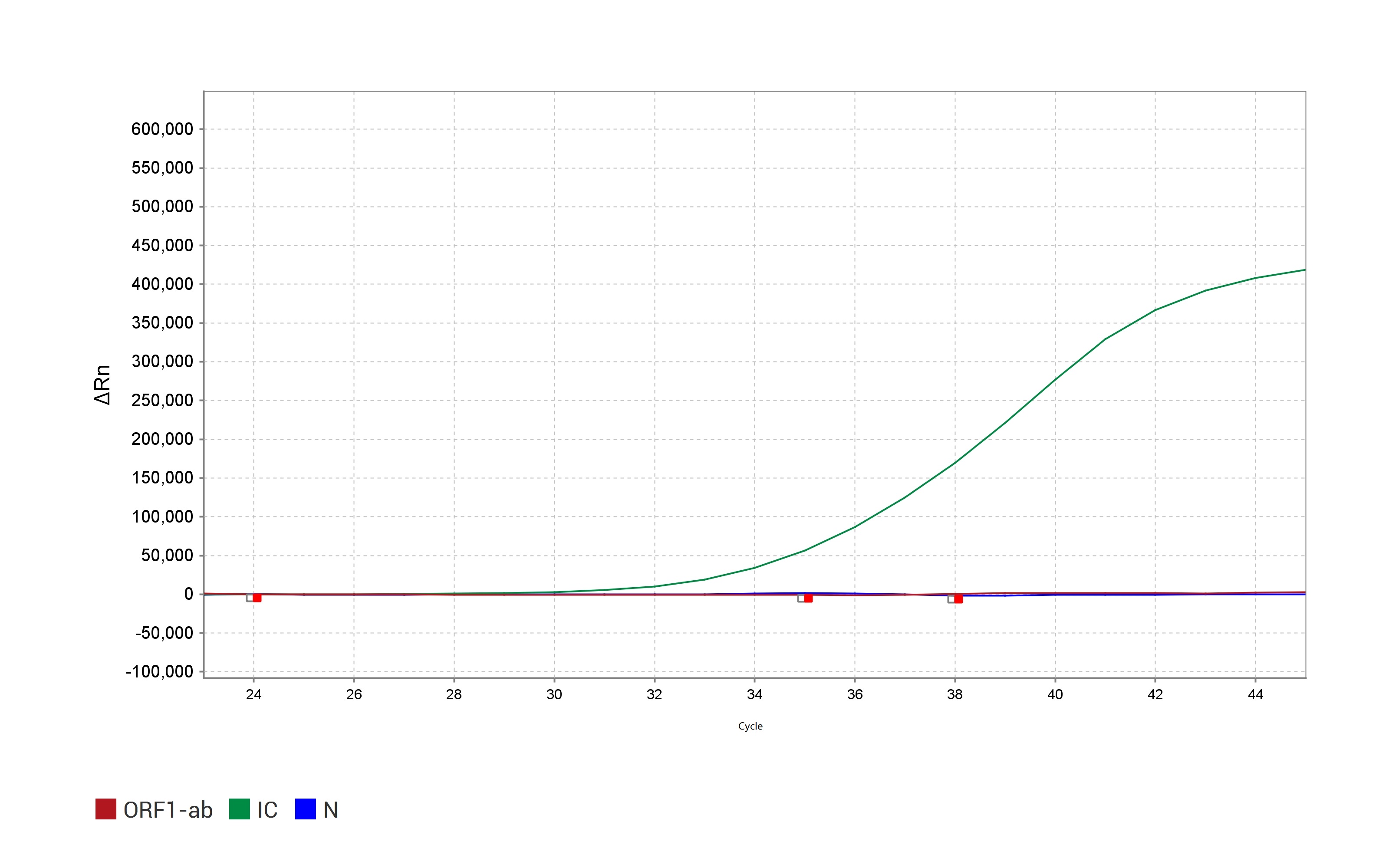

Supplement: S1 File — (ZIP) [file pone.0286121.s001.zip › DNA amplification graphs English/intensive care unit Semi-contaminated Hand sanitizer dispenser37.6.jpg]
