# Supplementary material for: SARS-CoV-2 RNA detection on environmental surfaces in COVID-19 wards
Source: PLoS One. 2023 May 25;18(5):e0286121. doi: 10.1371/journal.pone.0286121 (PMC10212077; doi:10.1371/journal.pone.0286121)
Supplement: Attachment — Submitted filename: Response to Reviewers.doc [file pone.0286121.s002.doc]

Dear editor

We feel great thanks for your professional review work on our article. As you are concerned, there are several problems that need to be addressed. According to your nice suggestions, we have made extensive corrections to our previous draft, the detailed corrections are listed below.

No 1. Compact abstract and add some results.

We have compacted abstract and added some results in the manuscript.

No 2. Discussion is very poor, improve it.

We have revised the discussion section in the manuscript.

No 3. Provide gel pictures as supporting information.

There is no gel picture in our experiment, but we provide DNA amplification graphs as supporting information.

No 4. Provide primer details used throughout work.

We provided photos of working in the file labeled ‘workflow’.

No 5. Provide systematic flow-chat of work for clear understanding.

We provided systematic flow-chat of work in the file labeled ‘workflow’.

No 6. What is the evidence of samples taken from hospital? The reviewer concerned about the sample collection. Please enlist all the samples taken for analysis from hospital and present their results in tabular form.

We enlisted all samples taken for analysis from hospital in file labeled ‘Sampling result statistics tabular form’.

No 7. Moreover, sample taken from surface of all wards immediately after disinfection spray or not?

All of our samples were taken one hour after disinfection.

No 8. Quantitative real-time reverse transcription 30 PCR (qRT-PCR) methods were used to confirm the presence of SARS-Cov-2 RNA. During analysis DNA amplification graphs generated as well as pictures must be reported to support the results.

We provided DNA amplification graphs in the file labeled ‘DNA amplification graphs’ to support the results.

No 9. What is the scenario of COVID test of staff working in represented wards of hospital?

All the scenario of COVID test of staff working in wards of hospital were negative.

No10. The reviewer also wonders about the statistical analysis of the article. It’s better to presents better in tabular form with appropriate tools but tables are missing in submitted manuscript.

We carefully reviewed our data and found some statistical errors. We corrected these statistical errors and provided the original results generated by SPSS in the file labeled ‘Statistical results’.

We deeply appreciate your consideration of our manuscript, and we look forward to receiving comments from the reviewers. If you have any queries, please don’t hesitate to contact me at the address below.

Thank you and best regards.

Yours sincerely,

Tao Zhao

Corresponding author:

Name: Tao Zhao E-mail: 15186613384@163.com
